# Signalling and Bioactive Metabolites from *Streptomyces* sp. RK44

**DOI:** 10.3390/molecules25030460

**Published:** 2020-01-22

**Authors:** Qing Fang, Fleurdeliz Maglangit, Linrui Wu, Rainer Ebel, Kwaku Kyeremeh, Jeanette H. Andersen, Frederick Annang, Guiomar Pérez-Moreno, Fernando Reyes, Hai Deng

**Affiliations:** 1 Marine Biodiscovery Centre, Department of Chemistry, University of Aberdeen, Meston Walk, Aberdeen AB24 3UE, UK; r01qf16@abdn.ac.uk (Q.F.); r01fm16@abdn.ac.uk (F.M.); r03lw15@abdn.ac.uk (L.W.); r.ebel@abdn.ac.uk (R.E.); 2 College of Science, Department of Biology and Environmental Science, University of the Philippines Cebu, Lahug, Cebu 6000, Philippines; 3 Marine and Plant Research Laboratory of Ghana, Department of Chemistry, University of Ghana, P.O. Box LG56 Legon, Accra, Ghana; kkyeremeh@ug.edu.gh; 4 Marbio, UiT—The Arctic University of Norway, N-9037 Tromsø, Norway; jeanette.h.andersen@uit.no; 5 Fundación MEDINA, Avda. del Conocimiento 34, 18016 Armilla, Granada, Spain; freddie.annang@medinaandalucia.es (F.A.); fernando.reyes@medinaandalucia.es (F.R.); 6 Instituto de Parasitología y Biomedicina “López-Neyra”, Consejo Superior de Investigaciones Científicas (CSIC) Avda. del Conocimiento 17, 18016 Armilla, Granada, Spain; guiomar@ipb.csic.es

**Keywords:** AHFA, methylenomycin, MMFs, signalling molecules, *Streptomyces* sp. RK44, anticancer, antimalaria

## Abstract

*Streptomyces* remains one of the prolific sources of structural diversity, and a reservoir to mine for novel natural products. Continued screening for new Streptomyces strains in our laboratory led to the isolation of *Streptomyces* sp. RK44 from the underexplored areas of Kintampo waterfalls, Ghana, Africa. Preliminary screening of the metabolites from this strain resulted in the characterization of a new 2-alkyl-4-hydroxymethylfuran carboxamide (AHFA) **1** together with five known compounds, cyclo-(L-Pro-Gly) **2**, cyclo-(L-Pro-L-Phe) **3**, cyclo-(L-Pro-L-Val) **4**, cyclo-(L-Leu-Hyp) **5**, and deferoxamine E **6**. AHFA **1**, a methylenomycin (MMF) homolog, exhibited anti-proliferative activity (EC_50_ = 89.6 µM) against melanoma A2058 cell lines. This activity, albeit weak is the first report amongst MMFs. Furthermore, the putative biosynthetic gene cluster (*ahfa*) was identified for the biosynthesis of AHFA **1**. DFO-E **6** displayed potent anti-plasmodial activity (IC_50_ = 1.08 µM) against *P. falciparum* 3D7. High-resolution electrospray ionization mass spectrometry (HR ESIMS) and molecular network assisted the targeted-isolation process, and tentatively identified six AHFA analogues, **7**–**12** and six siderophores **13**–**18**.

## 1. Introduction

*Streptomyces* have been, for decades, one of the most prolific sources of pharmacologically-active compounds, contributing for more than half of the naturally-derived antibiotics that are in clinical use today, such as chloramphenicol (from *S. venezuelae*) [1], fosfomycin (from *S. fradiae*) [2], clavulanic acid (from *S. clavuligerus*) [3], and avermectin (from *S. avermitilis*) [4]. In our efforts to discover natural products, we have isolated a new bacterial strain, *Streptomyces* sp. RK44 from the underexplored areas of Kintampo waterfalls, Ghana, Africa [5,6].

Chemical profiling of the RK44 extract using high-resolution electrospray ionization mass spectrometry (HR-ESIMS) and Global Natural Product Social (GNPS) molecular network (MN) [7] showed a cluster of low molecular weight metabolites with a characteristic 250 nm UV absorption, suggesting that they share a common chromophore skeleton. Database dereplication using AntiBase 2017 (WILEY) and other online databases (The Natural Products Atlas, ChemSpider) [8,9] revealed that these metabolites have not been previously described in the literature [10,11].

The isolation was carried out and afforded a new metabolite **1** (1.0 mg). Interpretation of the spectroscopic data demonstrated that **1** is 2-alkyl-4-hydroxymethylfuran-3-carboxamide (AHFA), a new methylenomycin furan (MMF) homolog which bears an amide moiety at C-3 instead of carboxylic acid [12] in the former. MMFs are signalling molecules or autoregulators identified in 2008 in the model actinomycete *Streptomyces coelicolor*, which induce methylenomycin antibiotic production [10]. Examples of the well-characterized autoregulators include acylhomoserine lactones (AHLs) in Gram-negative bacteria [13] and γ-butyrolactones (GBLs) in Gram-positive bacteria of the genus *Streptomyces* [14,15].

Genome mining revealed that the furan-type autoregulators may be widespread in *Streptomyces* species [10], however, only one class of MMFs has been identified to date [12]. The discovery of this new class of 2-alkyl-4-hydroxymethylfuran-3-carboxamide signalling molecule, AHFA **1** further expanded the chemical space of the under-explored class of furan-based autoregulators, and the MMF biosynthetic machinery. Furthermore, the presence of AHFA **7**–**10**, bearing various alkyl substituents at C-2 of the furan ring was also detected by high-pressure liquid chromatography (HPLC)-Ultraviolet (UV)-HRESIMS-GNPS analyses, but they were not isolated due to their trace quantities in the extract.

Along with AHFA **1**, we also isolated known compounds, cyclo-(L-Pro-Gly) **2**, cyclo-(L-Pro-L-Phe) **3**, cyclo-(L-Pro-L-Val) **4**, cyclo-(L-Leu-Hyp) **5**, and deferoxamine E **6**. Moreover, the MN analysis also identified the clusters corresponding to diketopiperazines **2**–**5**, and siderophores **6** and **13**–**18**.

## 2. Results and Discussion

A large-scale fermentation (6 L) of *Streptomyces* sp. RK44 in Modified Bennett’s broth [16] was performed for seven days (28 °C, 180 rpm). Subsequently, Diaion^®^ HP-20 (3 g/50 mL) was added to the culture broth and incubated overnight under the same culture conditions. The mixture was filtered under vacuum, after which the residue consisting of the mycelium and HP-20 resin was submerged in 100% methanol (3 × 500 mL) for 24 h. The methanol extract was then decanted and concentrated under reduced pressure to give a crude extract (5.8 g) which was then fractionated by solid-phase extraction (SPE) using Strata^®^ C18-E to give six fractions (S1–S6).

**Figure 1:**
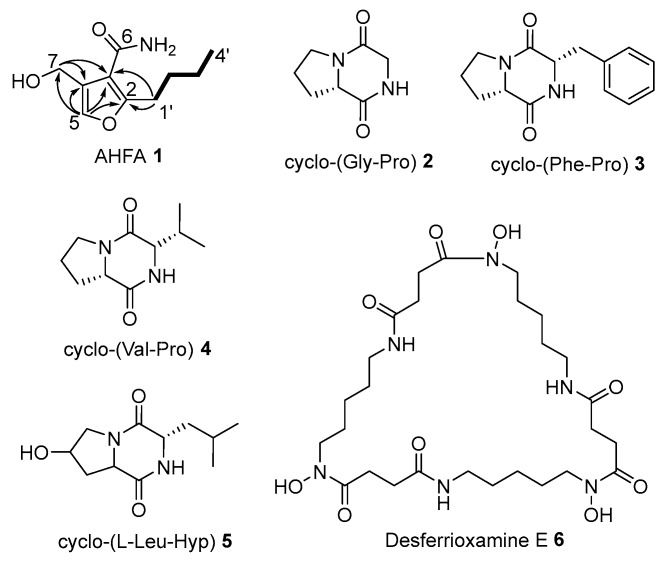
Isolated metabolites from *Streptomyces* sp. RK44. structures of which were elucidated by NMR. Structure of AHFA **1** with COSY (**―**) and key HMBC (**→**) correlations.

Dereplication of SPE extracts of RK44 using GNPS and a survey of databases (e.g., Dictionary of Natural Products, AntiBase, The Natural Products Atlas) suggested the presence of potentially new metabolites cluster in fraction S3. Consequently, S3 was selected for further purification using semi-preparative reversed-phase HPLC to yield **1** (1.0 mg), along with a number of diketopiperazines **2** (1.2 mg)**, 3** (2.0 mg)**, 4** (2.1 mg)**, 5** (2.3 mg), and the known siderophore, deferoxamine E **6** (3.0 mg) (Figure 1).

### 2.1. Structure Elucidation

The structures of the diketopiperazines (DKPs), cyclo-(L-Pro-Gly) **2**, cyclo-(L-Pro-L-Phe) **3**, cyclo-(L-Pro-L-Val) **4**, and cyclo-(L-Leu-Hyp) **5**, were determined by comparison of the UV spectra, molecular formulae, and a series of Nuclear magnetic resonance (NMR) spectra with literature data [17,18,19,20,21] (Figure S21–S24, Table S2). The configuration of **2**–**5** were determined by advanced Marfey’s analysis, suggesting that all the proteogenic amino acids are in L-configuration (Table S4). Compound **6** was identical to the reported siderophore, desferrioxamine E **6** [22,23,24] (Figure S22, Table S3).

Compound **1** was obtained as a yellow powder. The molecular formula of **1** (Figure 1) was established as C_10_H_15_NO_3_ by High-Resolution Electrospray Ionization Mass Spectrometry (HR-ESIMS) (calculated [M + H]^+^ = 198.1123; observed [M + H]^+^ = 198.1125; ∆ = −0.5047 ppm), indicating 4 degrees of unsaturation (Figure S1). The UV absorption maximum at 250 nm found in its spectrum is a characteristic feature of MMF molecules.

Analysis of the ^1^H, HMBC, and HSQC spectra revealed the presence of 1 sp^2^ methine, 4 methylenes, 1 methyl, and 4 quaternary carbons leading to the sub-formula C_10_H_12_ (Table 1). The ^1^H-NMR spectrum in CD_3_OD contained signals for 12 of the 15 protons accounted by the molecular formula, suggesting three exchangeable protons. These protons were observed in the DMSO-*d*_6_ spectrum at δ_H_ 6.53 (NH_2_) and δ_H_ 4.05 (OH). The remaining oxygen atom and the ^13^C chemical shifts (δ_C_ 162.1, 123.1, 139.1, 115.8), and the number of double bond equivalents suggested the presence of a furan core in the structure, which is supported by the heteronuclear multiple bond correlations (HMBC) from H-5 (δ_H_ 7.38, s) to C-2 (δ_C_ 162.1), C-3 (δ_C_ 115.8), and C-4 (δ_C_ 123.1).

Inspection of the ^1^H-^1^H COSY data identified one spin system from H-1′to H-4′, indicative of a butyl alkyl chain. The cross peak from H_2_-1′ (δ_H_ 2.95, t) to C-2 (δ_C_ 162.1) established the connectivity of the butyl alkyl group to the furan framework. The observed downfield signal at δ_H_ 4.52 (H_2_-7, s) suggested a hydroxymethylene group, and it was placed at C-4 based on the strong HMBC from H-7 (δ_H_ 4.52) to C-3 (δ_C_ 115.8), C-4 (δ_C_ 123.1), and C-5 (δ_C_ 139.1). The carbonyl carbon at C-6 (δ_C_ 171.6) and the N-H signal (δ_H_ 6.53, 2H) obtained in DMSO-*d*_6_ accounted for an amide moiety, and its connectivity was assigned to the remaining quaternary carbon C-3 (δ_C_ 115.8) of the furan ring.

Final structural analysis of **1** was confirmed by comparison with the spectroscopic data reported for MMF4 [12], which differs with **1** in the presence of a carboxylic acid in the former instead of the amide at position C-3. Compound **1** was therefore identified as 2-alkyl-4-hydroxymethylfruan carboxamide (AHFA).

MMFs share the common furan core in the structure, and the alkyl substituents at position C-2 of the ring can vary depending on which starter unit is incorporated during fatty acid biosynthesis [12,25]. Based on the HR ESIMS/GNPS analysis of the *Streptomyces* sp. RK44 extract, we have tentatively identified six AHFA **1** analogues, **7**–**12**, with various lengths in their carbon alkyl chains at C-2 of the furan ring (Figures S10–S16, Table S1). However, they could not be isolated due to their presence in the extract in minute amounts. AHFA **7**–**12** clustered together in the molecular network (MN) signifying that they share a common structural motif evident in the presence of the furan framework (Figure S10). Detailed analysis of the UV pattern, MS and MS^2^ fragmentation data showed that they exhibit the characteristic UV absorption at 250 nm of MMFs, and the alkyl chain substitution at position C2 of the ring, analogous to the MMFs that have been reported previously [12].

Further MN analysis also identified the clusters corresponding to the DKPs **2**–**5**, deferoxamines (DFO) B and E **6** and the 2.5kDa RIPP peptide (Figure S9). The observed molecular ion peaks of **13**–**18** matched with the known ions in the GNPS library and they clustered together in the network, suggesting that they could be potentially new siderophores. Moreover, their fragmentation pathway were proposed based on the observed MS/MS data (Figure S20). Characterization of the peptide is currently underway in our laboratory.

### 2.2. Proposed MMF Biosynthetic Gene Cluster and Pathway

**Figure 2:**
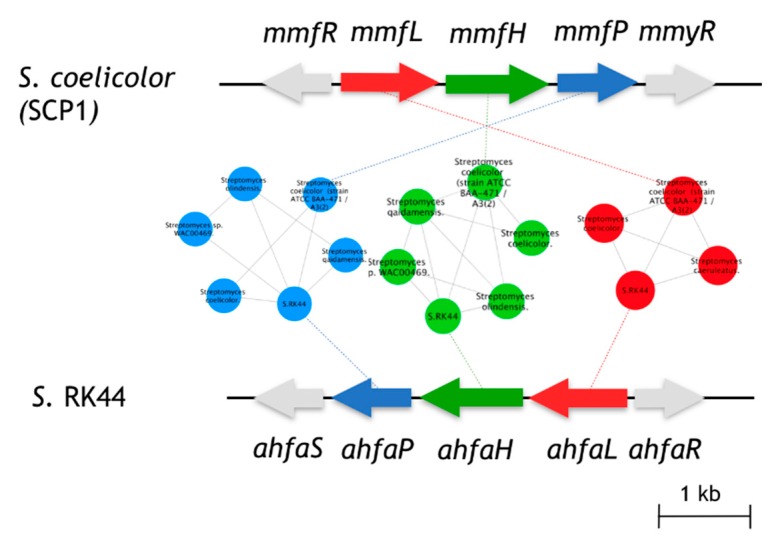
Comparison of the *ahfa* BGC in *Streptomyces* sp. RK44 with the *mmf* gene cluster on the SCP1 plasmid of *S. coelicolor* A3(2). Homologous genes are colored the same, and sequence identities between each of the encoded proteins are indicated in Table 2.

The biological function of AHFA **1** in the host cell is still unknown but is believed to be regulators of antibiotic biosynthesis in *Streptomyces* sp. RK44, analogous to the MMFs in *S. coelicolor* and GBLs in *Streptomyces* species [12,25]. Given the structural similarity of **1** with MMF4 [12], we predicted that **1** originated from an analogous biosynthetic pathway of MMF4. In silico analysis of the annotated RK44 genome using antiSMASH 3.0 [26] revealed one putative biosynthetic gene cluster, BGC (*ahfa*) that is likely to be involved in the biosynthesis of AHFA **1**. Pairwise comparison of the annotated proteins using BLAST [27] and the sequence similarity network (SSN) [28] between the *ahfa* cluster with the *mmf* BGC showed high amino acid identity, supporting its role in MMF homolog biosynthesis (Figure 2).

**Scheme 1:**
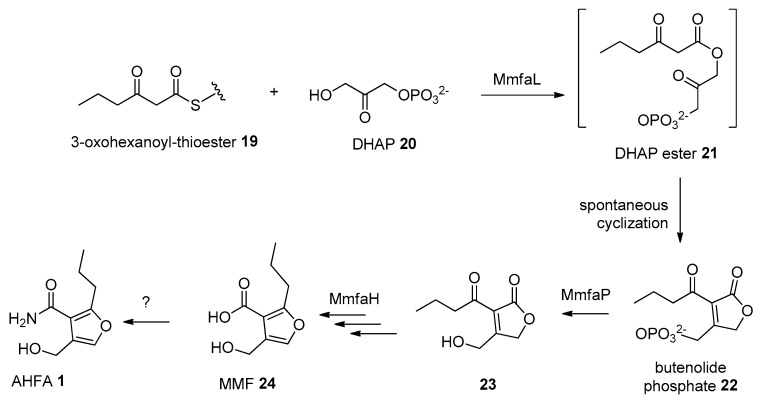
Proposed biosynthesis pathway of **1**.

The *ahfa* gene cluster spans about 4.5 kb of genomic DNA and contains three biosynthetic genes (*ahfa*L, *ahfa*P, *ahfa*H) that are likely involved in the biosynthesis of AHFA **1** in *Streptomyces* sp. RK44 (Table 2, Scheme 1). The incorporation of stereospecifically ^13^C-labeled glycerols into the MMFs suggested that they are biosynthesized via a butenolide phosphate intermediate **22** [25], derived from the MmfL-catalysed condensation of coenzyme A β-ketothioesters **19** in fatty acid biosynthesis with dihydroxyacetone (DHAP) **20**. The isotope-labelled studies indicated that the hydroxymethyl group in MMFs originated from the hydroxymethyl of DHAP, suggesting an analogous role of AhfaL to the AfsA in the biosynthesis of A-factor [29], in contrary to the alternative roles proposed by Sello and colleagues (2009) [30]. AhfaL which encodes for butenolide synthase in RK44 with 53% identity to *mmfL* and 42.0% similarity to *afsa* is proposed to catalyse a similar condensation reaction between 3-oxohexanoyl-thioester **19** and DHAP **20** to yield **21**, then **22**. AhfaP encoded for phosphatase and has 58% identity to MmfP is likely responsible for the dephosphorylation of butenolide **22** to form **23**, followed by rearrangement to **24** catalysed by the flavin-dependent monooxygenase, AhfaH. The formation of the furan ring in **1** and other AHFA analogues remains elusive.

Furthermore, two putative genes, ahfaR and ahfaS which encode for TetR/AcR transcriptional repressor showed high amino acid identities to MmfR and MmyR (46/62, WP_011039544.1; 62/75, WP_011039548.1) in *S. coelicolor* [31], respectively, were likely responsible for the antibiotic regulation in RK44.

The deduced function of ahfa*L*,*H*,*P* genes, based on their homology to autoregulatory genes (*mmf* or *afs*) of known function, supports the structure and the probable role of AHFA **1** as a signalling molecule.

### 2.3. Biological Test

**Figure 3:**
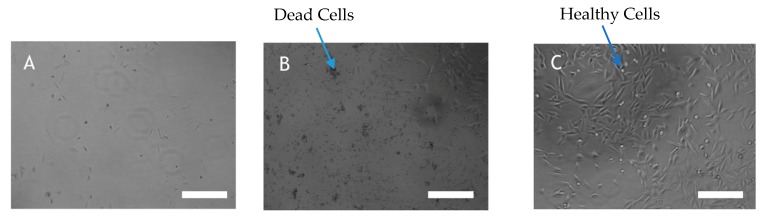
(**A**) A2058 cell treated with 50µg/mL staurosporine (**B**) A2058 cell treated with 50µg/mL AHFA 1 (**C**) A2058 cell treated with culture media. Cell viability was quantified by Aqueous One Solution Reagent by a colourimetric method for determining the number of viable cells in proliferation or cytotoxicity assays. A2058 cells were left untreated or were treated with AHFA **1** for 72 h and morphological changes of the cells were observed by light microscope (Scale bar 26 μm).

AHFA **1** inhibited proliferation and viability of human A2058 melanoma cells and induced anoikis (EC_50_ = 89.6 µM) (Figure 3B) compared to control (treated with culture media alone, Figure 3C). It is worth noting that, although weak, this activity is the first time to be reported amongst MMFs (Figure S23). Compounds **2**–**6** did not display any bioactivity at the highest concentration tested (50 µM).

Compounds **1**–**6** were also investigated for their anti-plasmodial activities against *Plasmodium falciparum* 3D7. Only **6** showed potent antimalarial activity (IC_50_ = 1.08 µM), and **1**–**5** did not display any bioactivity at the highest concentration tested (50 µM). The activity of **6** may result from the strong chelation of DFO-E **6** with iron from the culture medium of *P. falciparum*, causing iron deficit and finally cell death [32]. This observation was consistent with the reported antiplasmodial activities of siderophores in literature [33,34,35,36].

On the other hand, there are no significant antimicrobial activities observed in **1**–**6** against the Gram-negative pathogens, *Pseudomonas aeruginosa* ATCC 27853 and *Escherichia coli* ATCC 25922 at concentrations between 0–50 µg/mL.

## 3. Materials and Methods

### 3.1. Fermentation

*Streptomyces* sp. RK44 was isolated from the soil near Kintampo waterfall, Ghana, Africa. The soil isolate was streaked on an ISP2 agar plate (yeast extract 4 g, malt extract 10 g, glucose 4 g, 15 g agar, in 1L H2O) to get pure single colonies. One single colony was inoculated into 50 mL of Modified Bennett’s liquid medium (glycerol 10.0 g, Oxoid yeast extract 1.0 g, Bacto-Casitone 2.0, Lab-Lemco 0.8 g in 1000 mL MilliQ H_2_O), and incubated for three days at 28 °C with shaking at 180 rpm (Incu-shake FL16-2). This seed culture was used to inoculate 6.0 L of MB broth (1:100) in 12 baffled flasks (Corning^TM^ polycarbonate flasks), each containing autoclaved MB medium (500 mL) and plugged with Fisherbrand™ polyurethane foam stoppers (Fisher Scientific, UK). The cultures were fermented for seven days under the same condition as the seed culture. On day 7, Diaion^®^ HP-20 (3 g/50 mL) was added to the fermentation culture under sterile conditions. The flasks were left in the same shaking temperature and conditions for 16–22 h. The culture broth was filtered under vacuum (Buchi pump V100, UK), and the HP-20 resin was washed with MilliQ water and extracted thrice with 100% methanol (Fisher Chemical HPLC grade). All the methanol extracts were combined, concentrated under reduced pressure (Buchi Rotavapor R200, BUCHI, UK), and subjected to High-resolution Electrospray Ionization Liquid Chromatography Mass Spectrometry (HRESI-LC-MS, Thermo Scientific, UK) analysis.

### 3.2. Molecular Network Analysis

MSConvert software (3.0, Proteowizard, CA, US) was used to convert LCMS.RAW data to the mzXML format files. MZmine was used to preprocess the data and exported to the GNPS platform for molecular networking data analysis [37]. The GNPS analysis was achieved through the GNPS data analysis workflow using the spectral clustering algorithm [38]. Data analysis was performed using the following settings: parent mass tolerance 0.02 Da, ion tolerance 0.02 Da, minimum pairs cosine 0.7, minimum matched peaks 6, network TopK 10, minimum cluster size 2, and maximum connected component size 100. The spectral library matching was performed with a similar cosine threshold and minimum matched peaks. The spectral networks were imported into Cytoscape 3.6.1 and visualized using the force-directed layout [39].

### 3.3. Spectroscopic Analysis

HR-ESI-LC-MS was obtained using an LTQ Orbitrap LC-MS system (Thermo Scientific, UK) coupled to a Thermo Instrument HPLC system (Accela PDA detector, Accela PDA autosampler and Accela Pump) on a positive ESI mode (30,000), MS/MS resolution 7500, C18 (Sunfire 150 × 46 mm column). 0.1% formic acid in water and 0.1% formic acid in acetonitrile was used for reverse-phase separation using a gradient from 0–100% in 25 min. The instrument parameters were set as following: Capillary voltage 45 V, spray voltage 4.5 kV, capillary temperature 200 °C, auxiliary gas flow rate 10–20 arbitrary units, sheath gas flow rate 5 arbitrary units, mass range 150–2000 amu (maximum resolution 30,000×), MS scan 150–2000Da coupled with an automated full dependent MS-MS scan.

1D and 2D NMR data were obtained on a Bruker AVANCE III HD 600 MHz (Ascend^TM^14.1 Tesla, UK) with Prodigy TCI^TM^ cryoprobe at 298 K in DMSO-*d*_6_ and CD_3_OD (Goss Scientific). Trimethylsilane (TMS) was used as an internal standard.

### 3.4. Fractionation of the Extract

Fractionation of the crude methanol extract was achieved by using Strata^®^ C18-E solid-phase extraction (SPE) (55 µm, 70 Å, 20 g/60 mL) cartridges. The SPE column was initially washed with two column volumes (CV) of methanol and MilliQ water separately, and then, the crude sample was loaded onto the column. The column was then eluted stepwise with solvent mixtures of decreasing polarity (8CV/solvent mixture): Milli-Q H_2_O, 25% MeOH, 50% MeOH, 75% MeOH, 100% MeOH, and 100% MeOH with 0.05% trifluoroacetic acid (Acros Organics). The eluents were collected separately and labelled as fractions S1–S6. All the fractions were concentrated under reduced pressure (Buchi Rotavapor R200) and subjected to HR-ESI-MS analysis.

Mass spectrometry (MS) analysis was carried out in all the fractions (S1–S6) to target the MMF signalling molecules. Analysis of the HRESI-LC-MS data of fraction S3 revealed seven resolved peaks with [M + H]^+^ ions at m/z 184.0967, 198.1123, 212.128, respectively. Dereplication of these masses was achieved using AntiBase (2017), indicating that these molecules were previously unreported but might share a similar furan core as MMF molecules. Thus, fraction S3 was selected for further fractionation by reversed-phase semi-prep (C18 ACE 10 µM 10 × 250 mm column) HPLC (Agilent 1260 Infinity). The purification was carried out using a linear gradient from 20% H_2_O: MeOH (95:5) to 100% MeOH for 28 min with a solvent flow rate of 1.5 mL/min, to yield AHFA **1** (1.0 mg). Six other furan derivatives, **7**–**12** were identified in the same fraction by HRESI-LC-MS and molecular network analyses (Figure S9, Table S1). However, these molecules could not be isolated due to their presence in trace quantities in the extract. Compound **2**–**6** were isolated in the fraction S2, using a linear gradient from 10% H2O: MeOH (95:5) to 100% MeOH for 28 min with a solvent flow rate of 1.5 mL/min.

Along with **1**, we also isolated cyclo-(L-Pro-Gly) **2**, cyclo-(L-Pro-L-Phe) **3**, cyclo-(L-Pro-L-Val) **4**, cyclo-(L-Leu-Hyp) **5**, and deferoxamine E **6**.

AHFA **1**: 1.0mg; pale yellow powder. UV (CH_3_OH): 250 nm; ^1^H, ^13^C-NMR data, see Table 1; HRESIMS (positive mode) *m*/*z* calculated [M + H]^+^ = 198.1125; observed [M + H]^+^ = 198.1123; ∆ = −0.5050 ppm.

cyclo-(L-Pro-Gly) **2**: 1.2 mg; white solid; ^1^H, ^13^C-NMR data, see Figure S21, Table S2; HRESIMS (positive mode) *m*/*z* calculated [M + H]^+^ = 155.0815; observed [M + H]^+^ = 155.0821; ∆ = 2.870 ppm.

cyclo-(L-Pro-L-Phe) **3**: 2.0 mg; pale yellow solid; ^1^H, ^13^C-NMR data, see Figure S22, Table S2; [α]^25^_D_ = −17.2 (*c* 1.0, MeOH); HRESIMS (positive mode) *m/z* calculated [M + H]^+^ = 245.1285; observed [M + H]^+^ = 245.1276; ∆ = −2.631 ppm.

cyclo-(L-Pro-L-Val) **4**: 2.1 mg; pale yellow solid; ^1^H, ^13^C-NMR data, see Figure S23, Table S2; [α]^25^_D_ = −31.0 (*c* 1.0, MeOH); HRESIMS (positive mode) *m*/*z* calculated [M + H]^+^ = 197.1285; observed [M + H]^+^ = 197.1284; ∆ = −0.5070 ppm.

cyclo-(L-Leu-Hyp) **5**: 2.3 mg; yellow solid; ^1^H, ^13^C-NMR data, see Figure S24, Table S2; [α] ^25^_D_ = −11.6 (*c* 1.0, MeOH); HRESIMS (positive mode) *m/z* calculated [M + H]^+^ = 227.1390; observed [M + H]^+^ = 227.1392; ∆ = −0.7040 ppm.

Deferoxamine E **6**: 3.0 mg; pale yellow solid; ^1^H, ^13^C-NMR data, see Figure S25, Table S3; HRESIMS (positive mode) *m*/*z* calculated [M + H]^+^ = 601.3356; observed [M + H]^+^ = 601.3350; ∆ = −1.031 ppm.

### 3.5. Advanced Marfey’s Experiment

Compounds **2**–**5** (0.5mg) were hydrolysed in 6 N HCl (1 mL) for 17 h at 115 °C. The hydrolysate was evaporated to dryness, dissolved in H_2_O (100 µL), and then, treated with 1 M NaHCO_3_ (20 µL), and FDLA, fluorodinitrophenyl-5-L-leucine amide (1% solution in acetone, 100 µL). The mixture was incubated at 40 °C for 1 h. The reaction was neutralized by addition of 2 N HCl (20 µL) and evaporated to dryness at 40 °C under a stream of dry N_2_. The residue was dissolved in CH_3_OH (500 µL) and filtered (0.45 μm PTFE) prior to HPLC-Diode Array Detection (DAD) analysis. The separation was carried out using gradient elution CH_3_CN/H_2_O with 0.1% TFA (from 0% to 100% for 30 min, flow rate 1.0 mL/min, UV detection λ_max_ 340 nm).

The standard amino acids were derivatized with FDLA and analyzed by HPLC-DAD in the same manner as the test compounds.

### 3.6. Genome Sequencing of Streptomyces sp. RK44

The genome sequencing of *Streptomyces* sp. RK44 and the sequencing assembly service were provided by BGI-Shenzhen, China. A 300 bp paired-end genomic library of RK44 was prepared for Illumina sequencing. A total of 11,820,095 bp were obtained and assembled by SOAP denovo software. The assembled genome was submitted to the RAST server for annotation [40]. The open reading frames (ORFs) of the *ahfa* gene cluster was identified using the key enzymes encoded in the known *mmf* gene cluster as a sequence query. The 16s rDNA sequences and the *ahfa* cluster has been deposited in the NCBI (MN906804).

**Figure 4:**
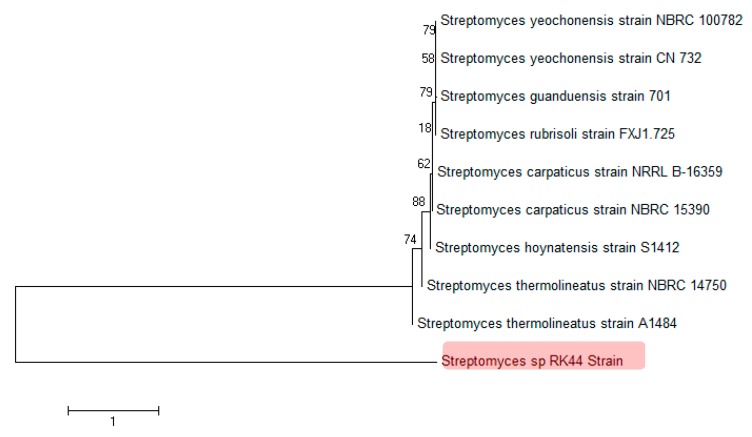
Phylogenetic analysis of 16S rDNA sequences of *Streptomyces* sp. RK44 and other known *Streptomyces* species, indicating that *Streptomyces* sp. RK44 is a new *Streptomyces* species.

Based on the partial 16s DNA gene sequence analysis it was identified that the strain belongs to the class *Streptomyces* and was most closely related to three strains with 82.49% sequence similarity: *Streptomyces hoynatensis* S1412, *Streptomyces carpaticus* NRRL B-16359, *Streptomyces yeochonensis* NBRC 100782. The strain formed a well-separated clade in the genus *Streptomyces* (Figure 4) based on the 16S rRNA gene analysis, indicating that *Streptomyces* sp. RK44 *is a* new *Streptomyces* species (MEGA 7, Neighbor-Joining method) [41,42]. The optimum pH for growth was determined to be 7.2. The DNA G+C content of the strain was determined to be 71.4 mol%.

### 3.7. Sequence Similarity Network

The sequence similarity network (Figure 2) of the biosynthetic gene clusters of *Streptomyces* sp. Rk44 and *Streptomyces coelicolor* A3(2) was constructed by all-to-all BLASTP comparison of sequences found in strains containing essential biosynthetic genes involved in the MMF and AHFA biosynthesis. Each node represents a gene and each edge represents the BLASTP pairwise comparison (E-value > 1 × 10^−10^) between two gene sequences. The results generated were visualized using Cytoscape 3.6.1. The genes, *mmfL*, *H*, *P*, are coloured with red, green, and blue, respectively. Other genes in the clusters were coloured with grey [28].

### 3.8. Anti-Proliferative/Cytotoxicity Test

The anti-proliferative activity of AHFA **1** was tested on the human melanoma A2058 cancer cell line (American Type Culture Collection (ATCC) CRL-11147^TM^, Manassas, VA, USA). Cell lines were seeded in 96-well-microtitre plates (Nunc, Thermo Fisher Scientific, NY, USA) at 2000 cells/well in Dulbecco’s Modified Eagle Medium (DMEM, Thermo Fisher Scientific, USA) with fetal bovine serum (10%, FBS, Sigma Aldrich, USA) and gentamicin (10 µg/mL, Sigma Aldrich, USA). Cells were grown at 37 °C in a humidified atmosphere of 5% CO_2_ and maintained at low passage.

Toxicity assay was tested on the lung normal cell (ATCC CCL-171).Cell lines were seeded in 96-well-microtitre plates (Nunc, Thermo Fisher Scientific, CA, USA) at 4000 cells/well in DMEM with FBS (10%) and gentamicin (10 µg/mL). Cells were grown at 37 °C in a humidified atmosphere of 5% CO_2_ and maintained at low passage.

Cells were incubated for 24 h before AHFA **1** was added and thereafter incubated for 72 h. Cell viability was determined by a colorimetric 3-(4,5-dimethylthiazol-2-yl)-5-(3 carboxymethoxyphenyl)-2-(4-sulfophenyl)-2H-tetrazolium (MTS) assay. At the end of the exposure time, 10 µL Cell Titer 96^®^ Aqueous One Solution Reagent (Promega, Madison, WI, USA) was added to each well. Results were measured after 1 h at 485 nm. The dose-dependent response of AHFA **1** (100 ng/mL, 1, 2.5, 5, 10, 12.5, 25, and 50 µg/mL) was tested in triplicates.

### 3.9. Antibacterial Assay

Minimum inhibitory concentrations (MIC) were determined using a conventional broth dilution assay in accordance with standards recommended by the National Committee for Clinical Laboratory Standards (NCCLS) [43]. Gram-negative bacteria *Escherichia coli* (ATCC 25922) and *Pseudomonas aeruginosa* (ATCC 27853) were used as test organisms. All bacteria were cultured in Mueller–Hinton broth. The assays were performed in 96-well plates (Nunc, Thermo Fisher Scientific, NY, USA), wherein a 50 µL suspension (log phase) of actively growing bacteria was incubated overnight at 37 °C and then treated with 50 µL of the test extract (final concentration of 50 μg/mL, for the three biological triplicates). The negative control comprised the growth media and Milli-Q water, while the positive control consisted of the bacteria plus Milli-Q water. The absorbance was recorded after 24 h (OD_600_) in a Victor3 multilabel plate reader. The growth medium appeared clear in wells where the test compound prevented the growth or killed the bacteria; otherwise, it was cloudy. Activity threshold was set below 0.05 (OD_600_).

### 3.10. Plasmodium Falciparum 3D7 Lactate Dehydrogenase Assay

The antiplasmodial activities of **1**–**6** were assessed by lactate dehydrogenase assay using *Plasmodium falciparum* 3D7 as the test organism. The assay was performed according to the method previously described by Pérez-Moreno et al., 2016 [36], in triplicate using a sixteen points dose-response curve (1⁄2 serial dilutions) with concentrations ranging from 50 μM to 1.5 nM to determine the IC_50_ of the pure compounds. Chloroquine was used as the standard reference.

## 4. Conclusions

Herein, we report the discovery of AHFA **1** from the novel soil bacterium *Streptomyces* sp. RK44. The structure of **1** was deduced by analysis of the HRESIMS, UV, 1D, and 2D NMR, and identified as a new AHFA-type natural product. Seven AHFA analogues, **7**–**12** and siderophores **13**–**18** were tentatively identified in the extract of RK44 by HRESIMS and GNPS molecular network analyses. A putative AHFA biosynthetic pathway was proposed for **1**. AHFA **1** displayed antiproliferative activity against melanoma A2058 cells (EC_50_ = 89.6 µM). Albeit weak, this represents the first report of such activity amongst MMF molecules. Five other known metabolites, cyclo-(L-Pro-Gly) **2**, cyclo-(L-Pro-L-Phe) **3**, cyclo-(L-Pro-L-Val) **4**, cyclo-(L-Leu-Hyp) **5**, and deferoxamine E **6** were isolated and structurally characterized, one of which, DFO-E **6** displayed potent activity against *P. falciparum* 3D7 (IC_50_ = 1.08 µM).

## Figures and Tables

**Table 1:** NMR data of AHFA **1** in CD_3_OD and DMSO-*d*_6_ at 600 MHz and 298 K.

		CD_3_OD		DMSO-*d*_6_
POSITION	^13^C (ppm)	^1^H (Integral, Mult.)	^13^C (ppm)	^1^H (Integral, Mult.)
4′	12.7	0.92 (3H, t)	13.9	1.01 (3H, t)
3′	21.9	1.35 (2H, hep)	21.9	1.41 (2H, hep)
2′	29.8	1.63 (2H, pent)	29.9	1.68 (2H, pent)
1′	26.6	2.95 (2H, t)	26.4	3.03 (2H, t)
2	162.1	-	161.3	-
7	54.1	4.52 (2H, s)	54.1	4.53 (2H, s)
6	171.6	-	169.9	-
5	139.1	7.38 (1H, s)	138.9	7.64 (1H, s)
4	123.1	-	124.2	-
3	115.8	-	117.0	-
NH_2_	-	-	-	6.53 (2H, br)
OH	-	-	-	4.05 (1H, br)

**Table 2:** Deduced functions of open reading frames (ORFs) in *ahfa* biosynthetic gene cluster.

Protein	AA	Homologue (% Identity/% Similarity)	Predicted Encoded Function
AhfaR	210	MmyR (62/75) *S. coelicolor* A3(2) A factor receptor (30/48) *S. griseus*	TetR/AcR family transcriptional regulator
AhfaP	243	MmfP (46/58) *S. coelicolor* A3(2)	Phosphatase/Hydrolase
AhfaH	384	MmfH (58/66) *S. coelicolor* A3(2)	Oxidoreductase
AhfaL	334	MmfL (42/53) *S. coelicolor* A3(2) AfsA (32/42) *S. griseus*	Butenolide synthase/ A factor biosynthesis
AhfaS	216	MmfR (46/62) *S. coelicolor* A3(2)	TetR/AcR family transcriptional regulator

## Supplementary Materials

The supplementary materials are available online.
