# Supplementary material for: Signalling and Bioactive Metabolites from Streptomyces sp. RK44
Source: Molecules. 2020 Jan 22;25(3):460. doi: 10.3390/molecules25030460 (PMC7037778; doi:10.3390/molecules25030460)
Supplement: Supplementary file 1 [file molecules-25-00460-s001.pdf]

molecules

Supporting Information

Signalling and Bioactive Metabolites from *Streptomyces* sp. RK44

Qing Fang [1], Fleurdeliz Maglangit [1,2] Linrui Wu [1], Rainer Ebel [1], Kwaku Kyeremeh [3], Jeanette H. Andersen [4], Frederick Annang [5], Guiomar Pérez-Moreno [6], Fernando Reyes [5] and Hai Deng [1,*]

Contents:

Figure S1. HR-ESIMS of AHFA **1**
Figure S2. MS/MS fragmentation of AHFA **1**
Figure S3. MS/MS fragmentation pathway of AHFA **1**
Figure S4. ^{1}H-NMR of AHFA **1**
Figure S5. ^{13}C-NMR of AHFA **1**
Figure S6. HSQC of AHFA **1**
Figure S7. HMBC of AHFA **1**
Figure S8. COSY of AHFA **1**
Figure S9. GNPS Molecular network clusters corresponding to the AHFA **1** and analogs **7-12**, RIPP peptide, DKPs, and siderophores observed in the *Streptomyces* sp. RK44 extract
Table S1. HR ESIMS data of **1-12**
Figure S10. GNPS molecular network of AHFA cluster and Extracted ion chromatograms of **1,7-12**
Figure S11–16. HR-ESIMS of **7-12**
Figure S17–19. HR ESIMS/MS of AHFA **1, 9, 10**
Figure S20. Siderophore molecular network
Table S2. ^{1}H and ^{13}C-NMR of **2-5**
Figure S21–S24. ^{1}H-NMR of **2-5**
Table S3. ^{1}H and ^{13}C-NMR of Deferoxamine E **6**
Figure S25. ^{1}H-NMR of Deferoxamine E **6**
Table S4. Advanced Marfey's analysis of **2-5**
Figure S26. MIC curve of **1** against A2058 cancer cell line
Figure S27. MIC curve of **6** against *P. falciparum* 3D7
References

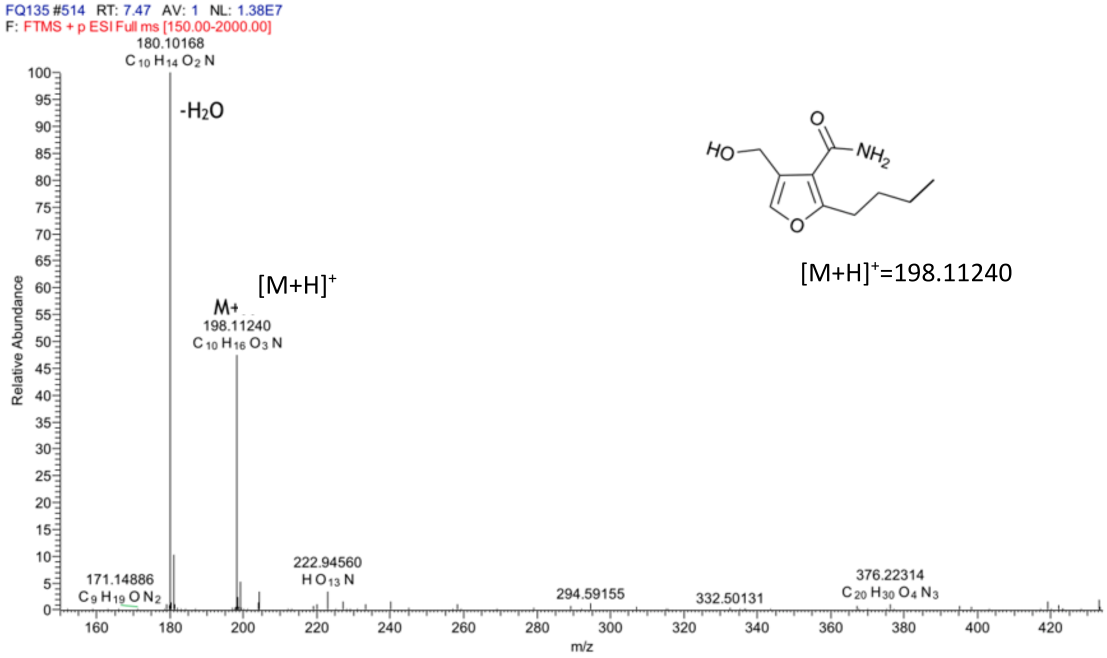

Figure S1. HR-ESIMS of AHFA **1.**

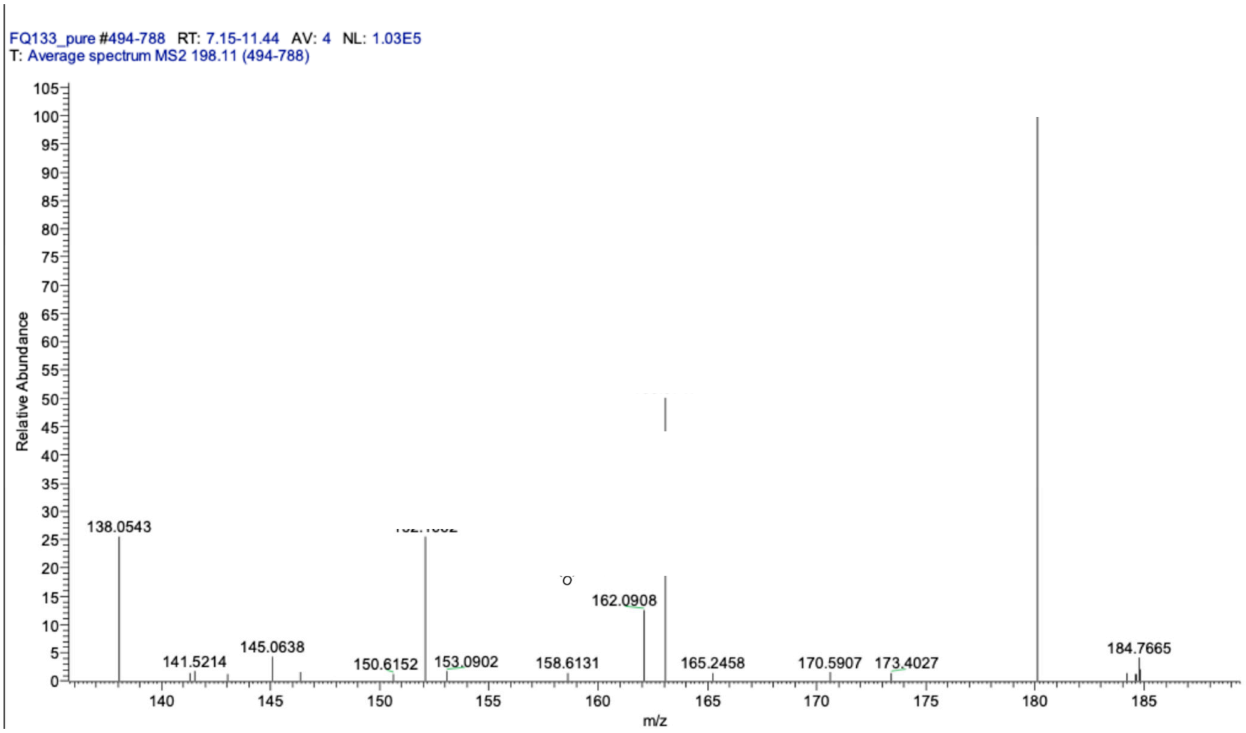

Figure S2. MS/MS fragmentation of AHFA **1.**

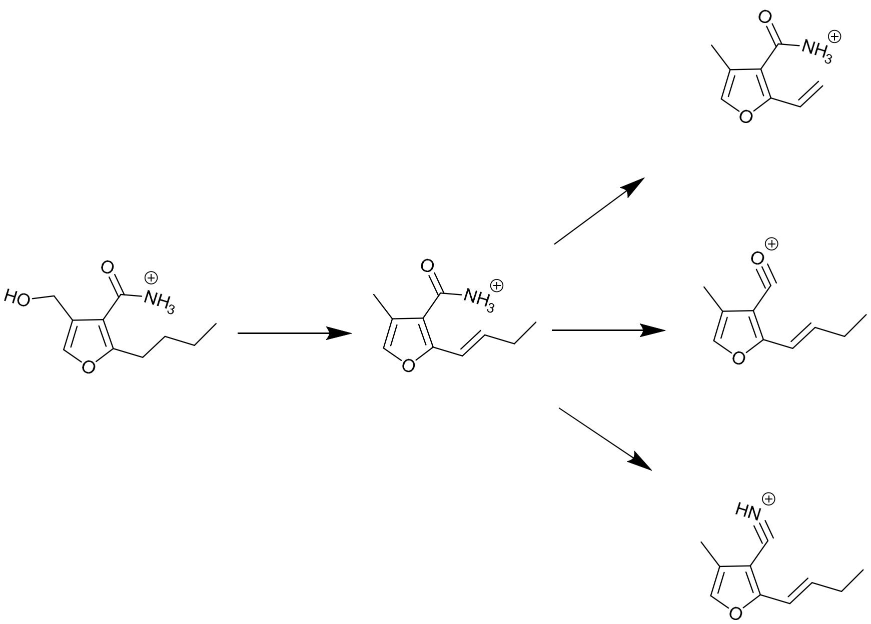

Figure S3. MS/MS fragmentation pathway of AHFA **1.**

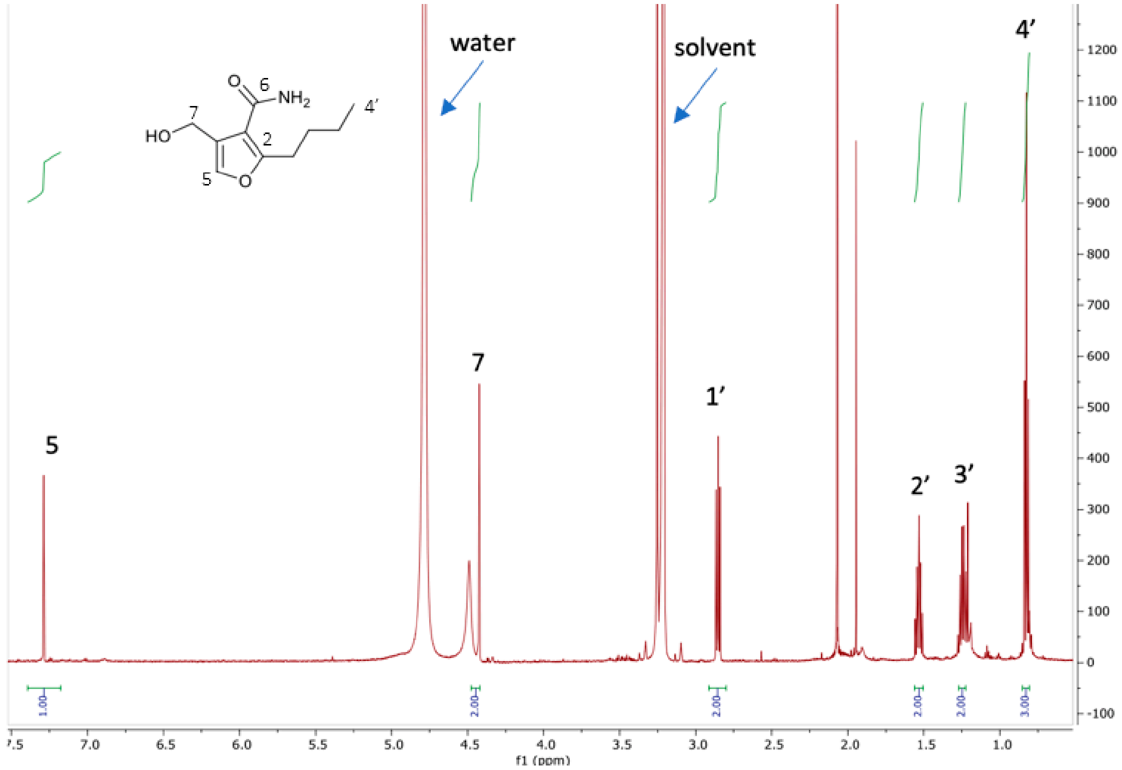

Figure S4. ^{1}H-NMR of AHFA **1** in CD_3OD (600MHz, 298K).

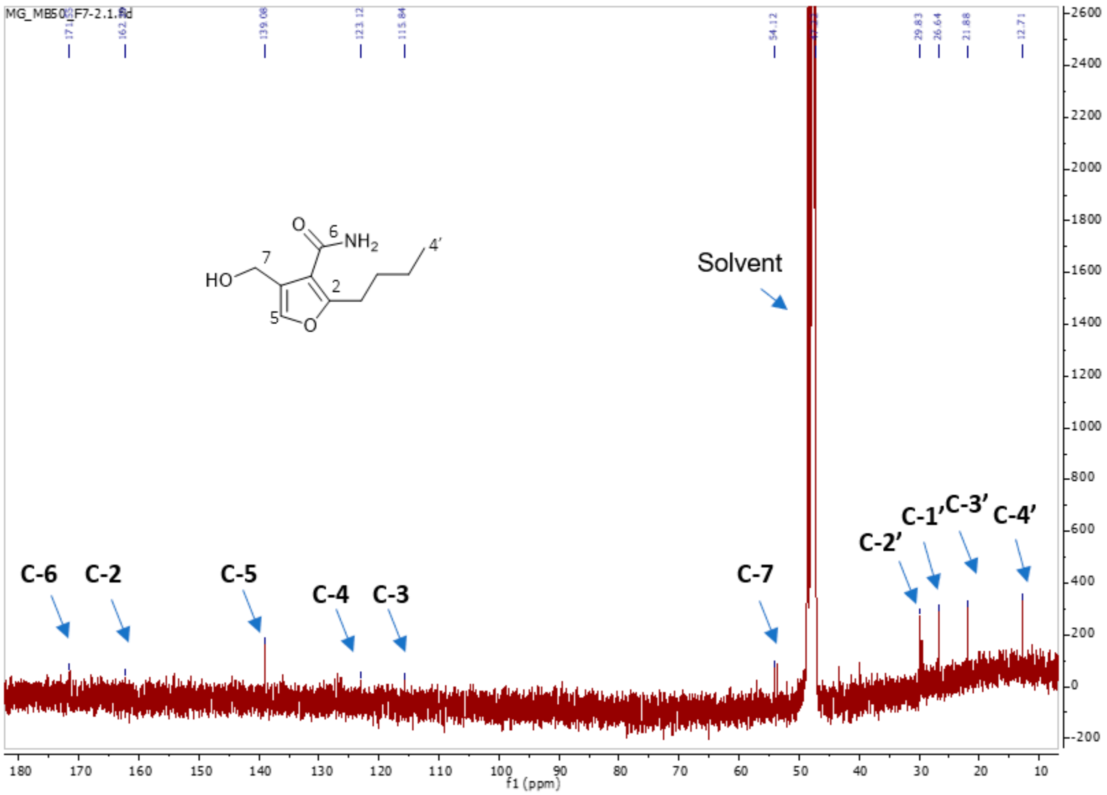

Figure S5. ^{13}C- NMR of AHFA **1** in CD$_3$D (600MHz, 298K).

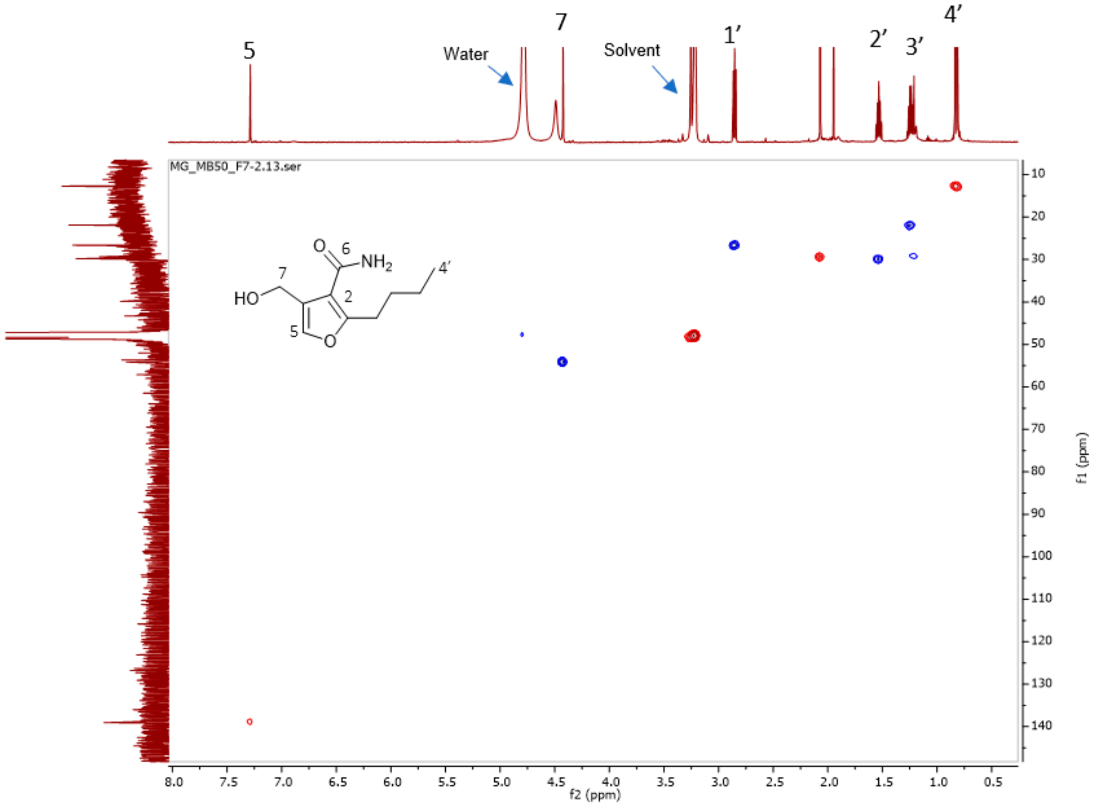

Figure S6. HSQC of AHFA **1** in CD$_3$OD (600MHz, 298K).

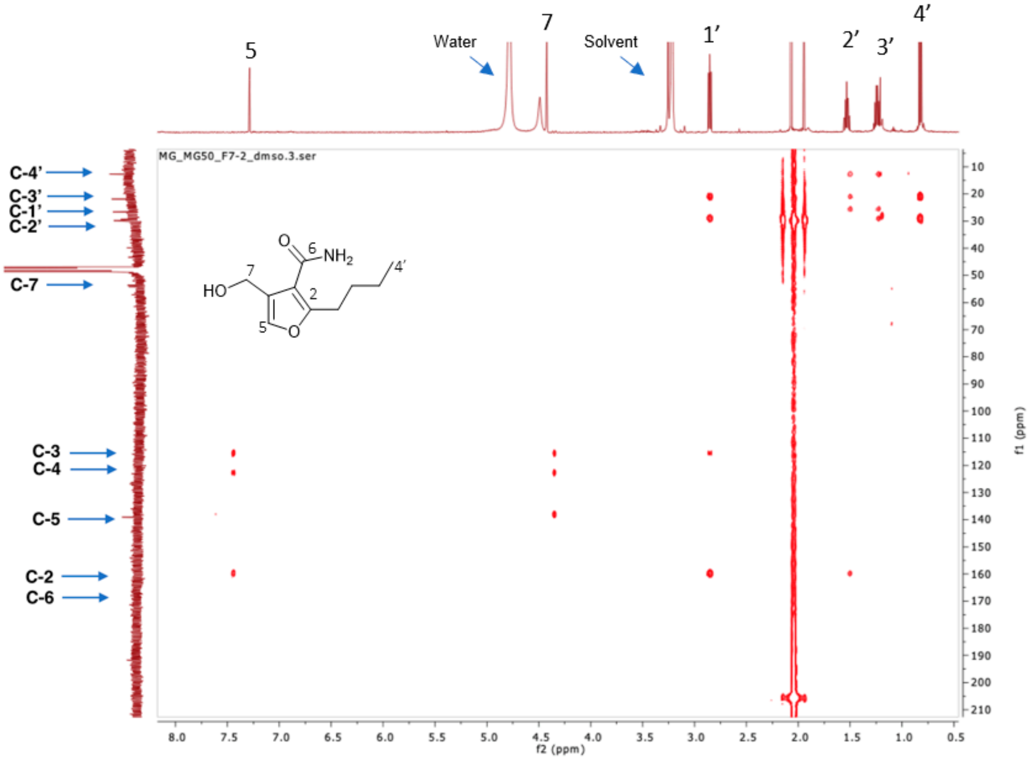

Figure S7. HMBC of AHFA **1** in CD_3OD (600MHz, 298K).

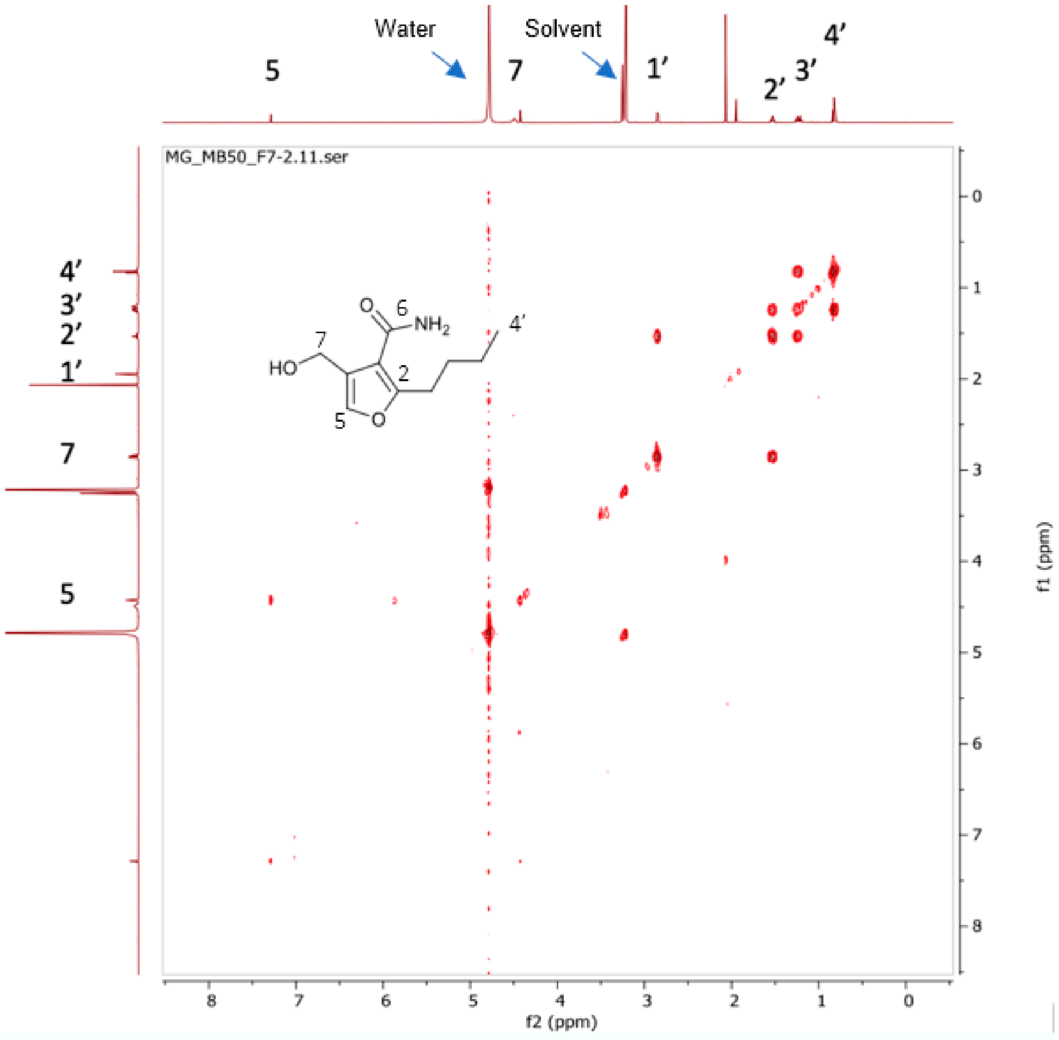

Figure S8. COSY of AHFA **1** in CD3OD (600MHz, 298K).

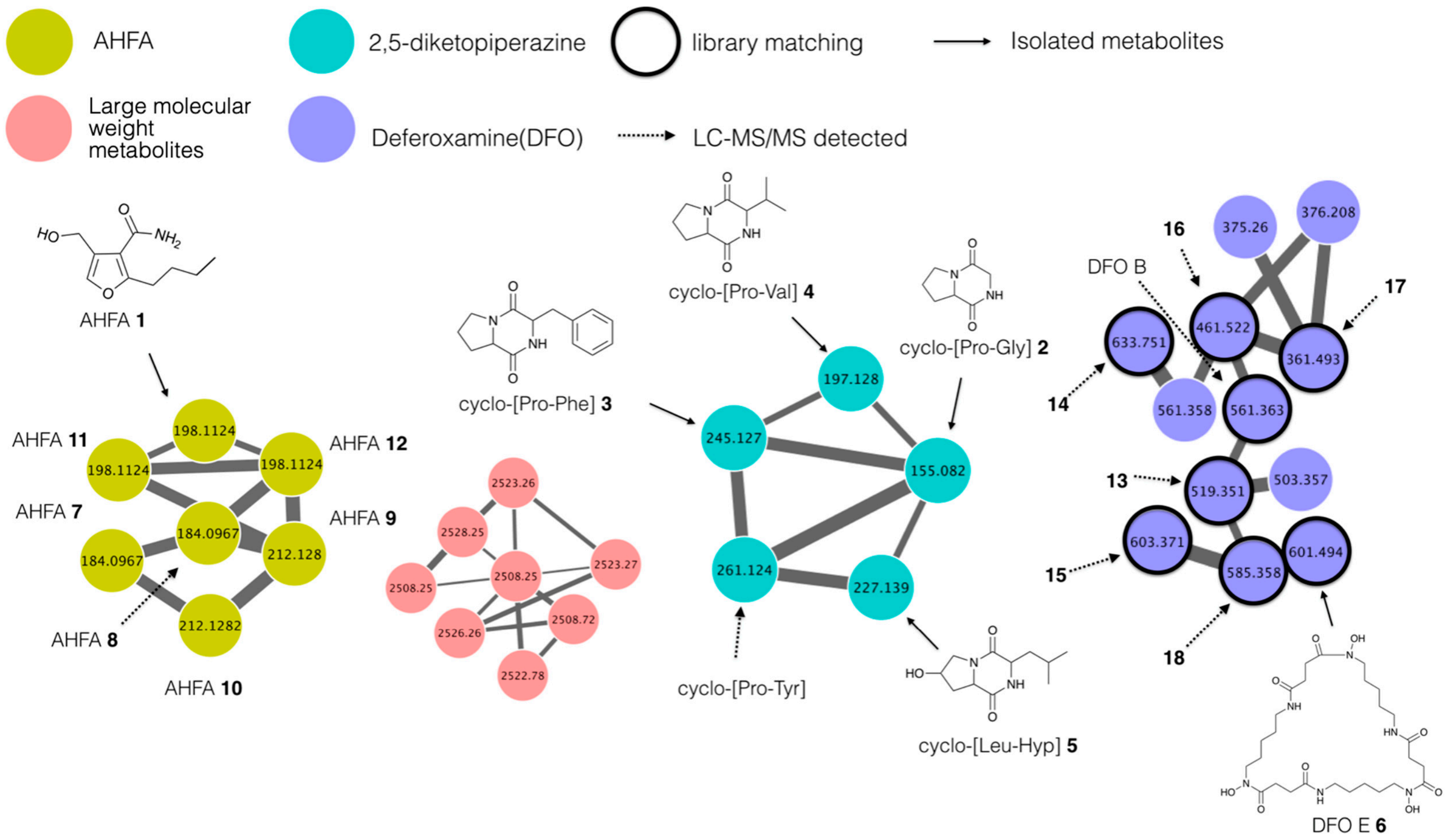

Figure S9. GNPS Molecular network clusters corresponding to the AHFA **1** and analogs **7-12**, Large molecular weight metabolites. (possible peptides), diketopiperazines, and siderophores, deferroxamine (DFO) B, DFO E **6** and **13-18** observed in the *Streptomyces* sp. RK44 extract.

Table S1. HR ESIMS data of **1-12.**

Name	MF	$[M+H]^+$ (calc.)	$[M+H]^+$ (obs)	Δ (ppm)
AHFA **1**	$C_{10}H_{15}NO_3$	198.1125	198.1124	-0.505
cyclo-[Pro-Gly] **2**	$C_7H_{10}N_2O_2$	155.0815	155.0821	2.870
cyclo-[Pro-Phe] **3**	$C_{14}H_{16}N_2O_2$	245.1285	245.1276	-2.631
cyclo-[Pro-Val] **4**	$C_{10}H_{16}N_2O_2$	197.1285	197.1284	-0.507
cyclo-[Pro-Hyp) **5**	$C_{11}H_{18}N_2O_3$	227.1390	227.1392	0.704
DFO-E **6**	$C_{27}H_{48}N_6O_9$	601.3556	601.3550	-1.031
AHFA **7**	$C_9H_{13}NO_3$	184.0968	184.0967	-0.540
AHFA **8**	$C_9H_{13}NO_3$	184.0968	184.0967	-0.540
AHFA **9**	$C_{11}H_{17}NO_3$	212.1281	212.1280	-0.470
AHFA **10**	$C_{11}H_{17}NO_3$	212.1281	212.1282	0.470
AHFA **11**	$C_{10}H_{15}NO_3$	198.1125	198.1124	-0.500
AHFA **12**	$C_{10}H_{15}NO_3$	198.1125	198.1124	-0.500

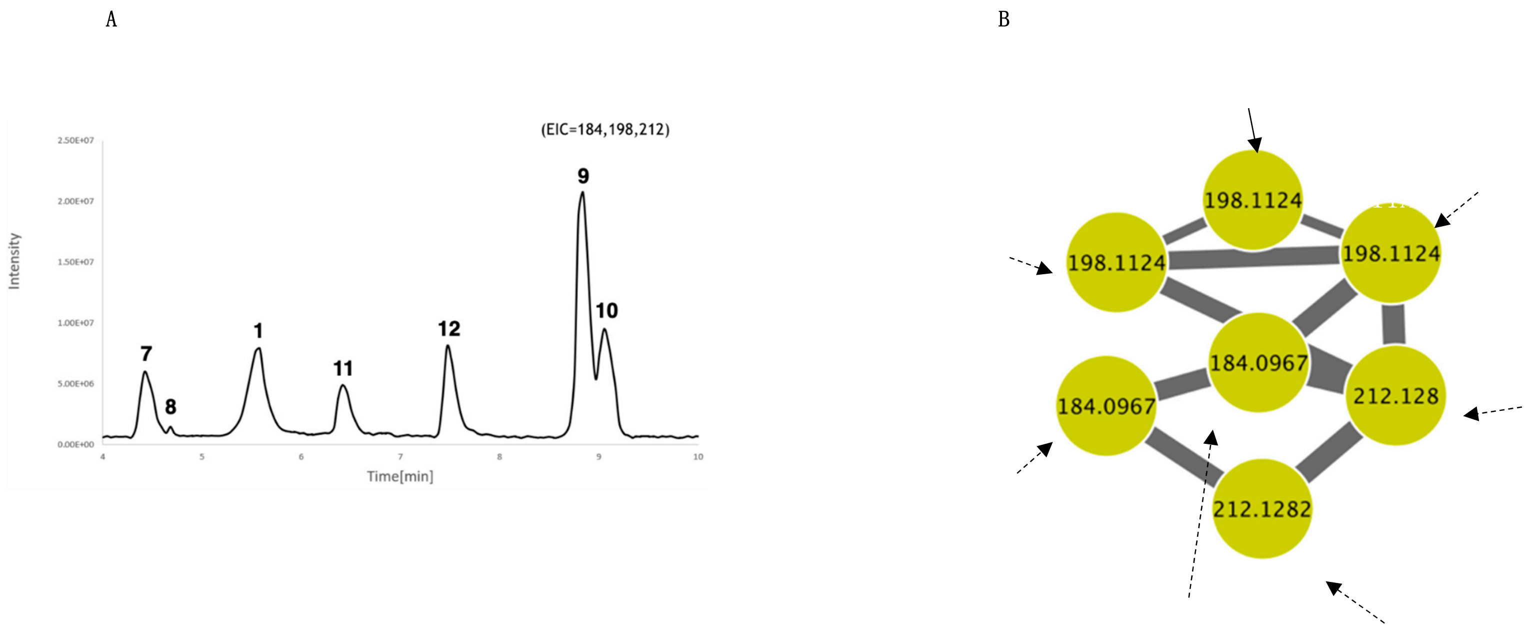

Figure S10. (**A**) Identification of the AHFAs produced by *Streptomyces* sp. RK44 by LC-MS. Extracted ion chromatograms (EICs) of AHFA **7, 8** (*m*/*z* =184), AHFA **1, 11, 12** (*m*/*z* = 198), AHFA **9, 10** (*m*/*z* = 212) (**B**) Spectral networks illustrating the results of the GNPS analysis with proposed structure based on their MS/MS fragmentation. The R group at C-2 of the furan ring are predicted based on the HR ESIMS and GNPS molecular network analyses, and by comparison to the MMF molecules in literature [1,2]. The alkyl chain of the pair **7** and **8**, **9** and **10, 11** and **12** are determined by LC retention time respectively. As branched chain compounds have shorter retention times than the corresponding straight-chain isomers, consistent with the literatures [1,2].

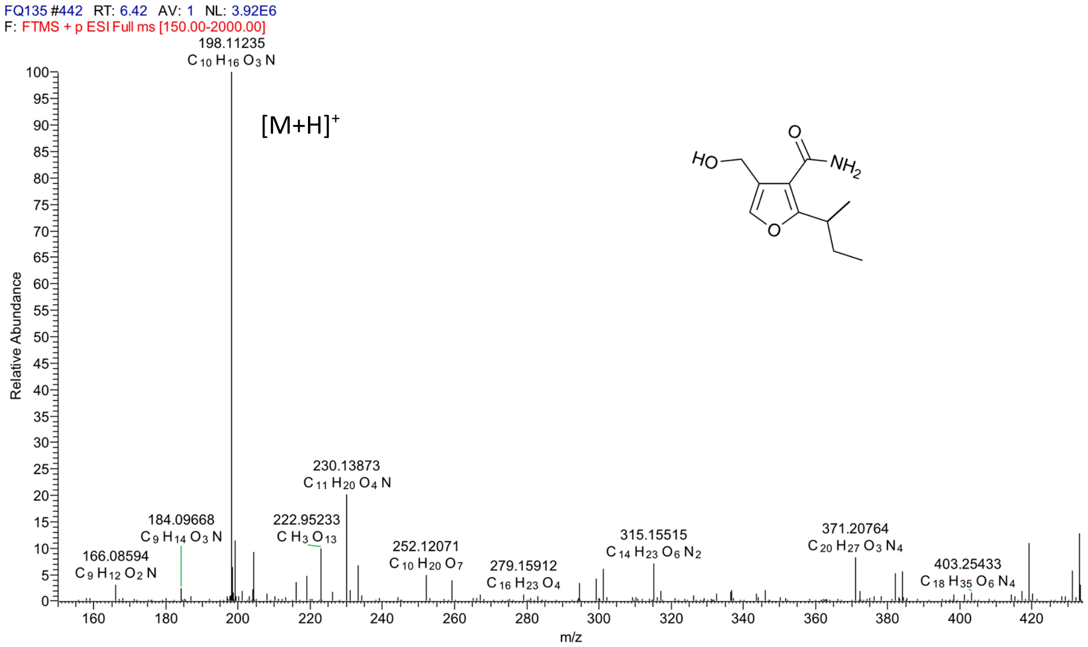

Figure S11. HR ESIMS of AHFA **7.**

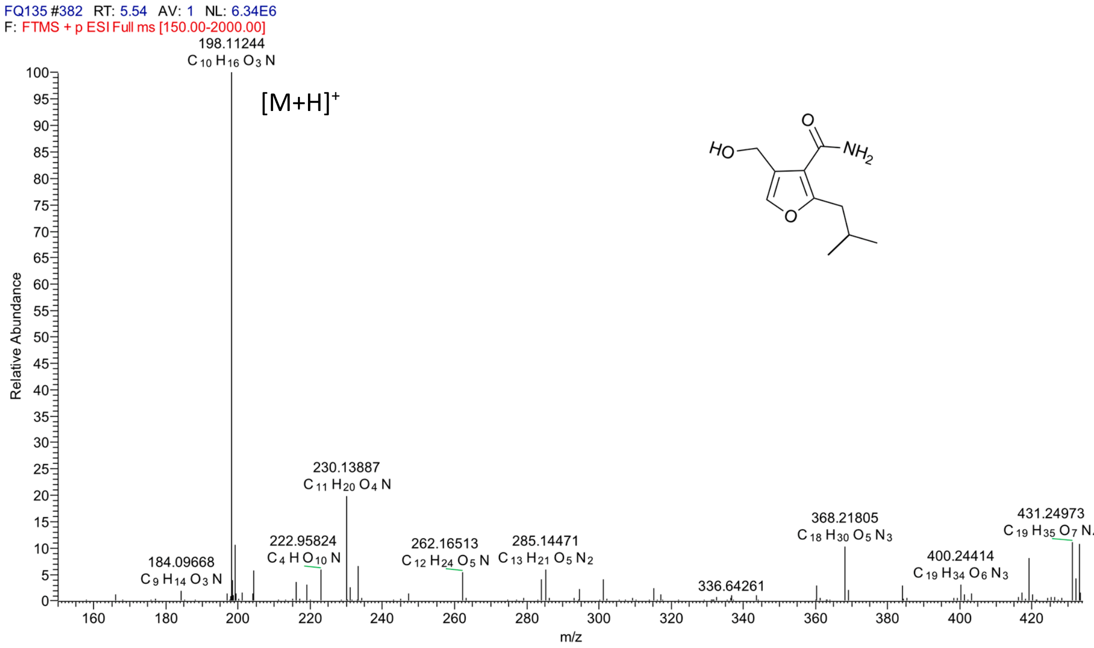

Figure S12. HR ESIMS of AHFA **8.**

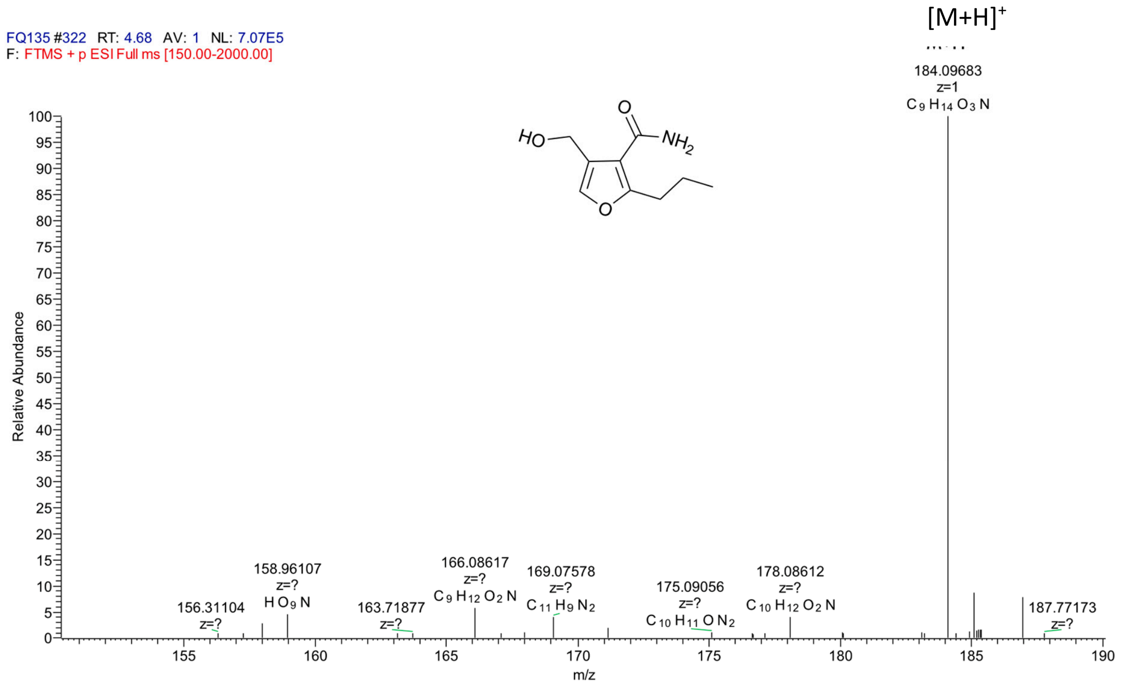

Figure S13. HR ESIMS of AHFA **9.**

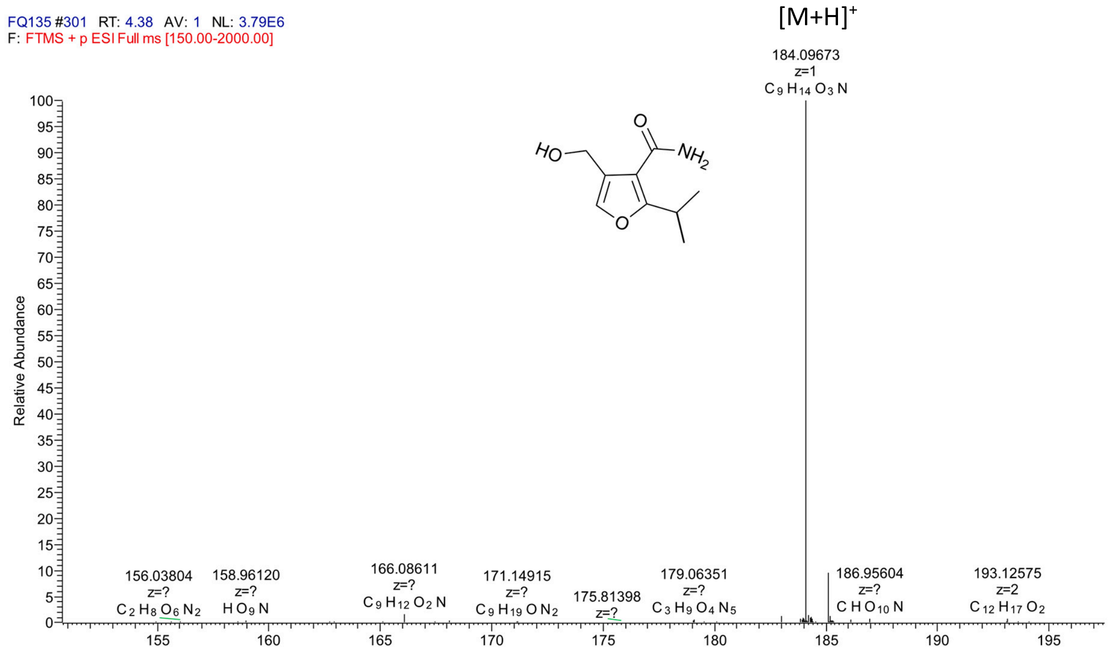

Figure S14. HR ESIMS of AHFA **10.**

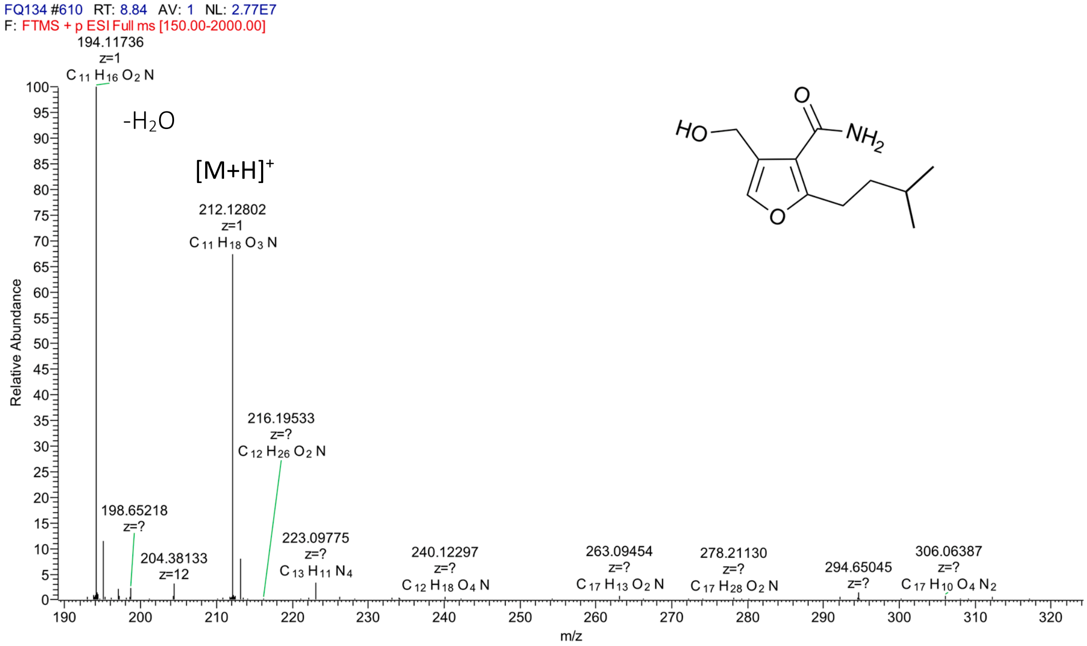

Figure S15. HR ESIMS of AHFA **11.**

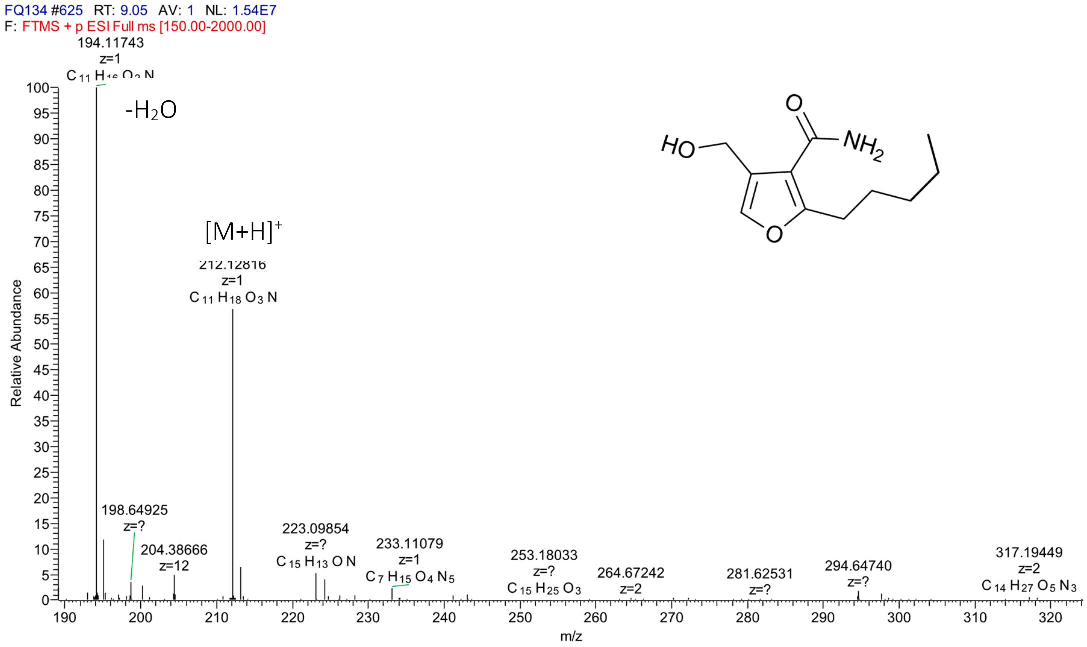

Figure S16. HR ESIMS of AHFA **12.**

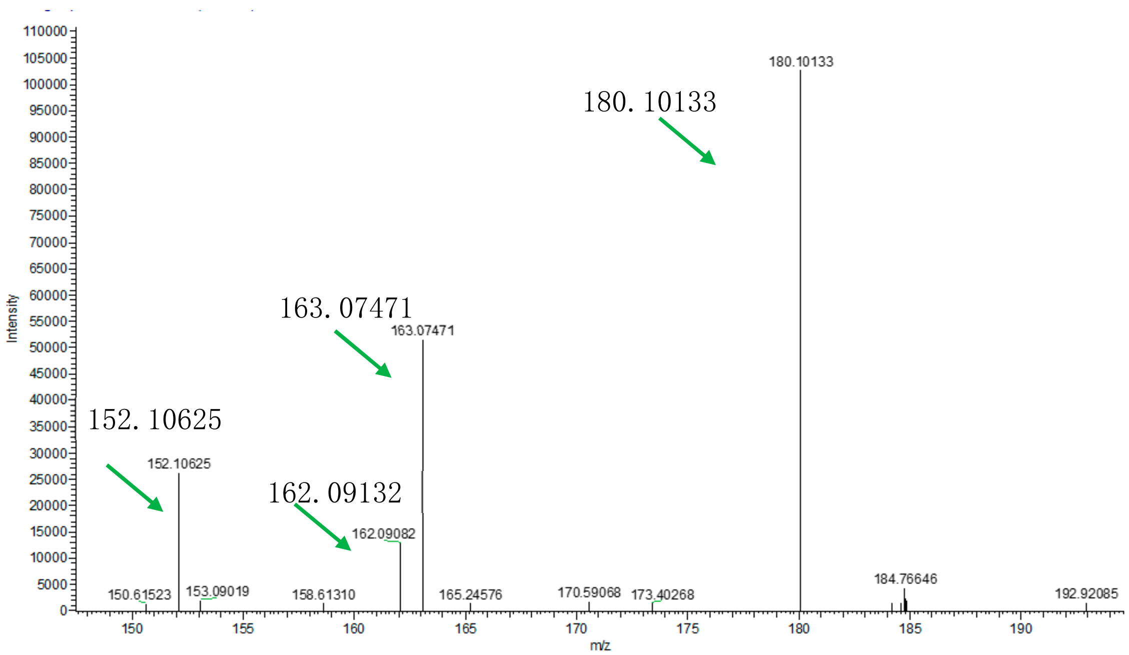

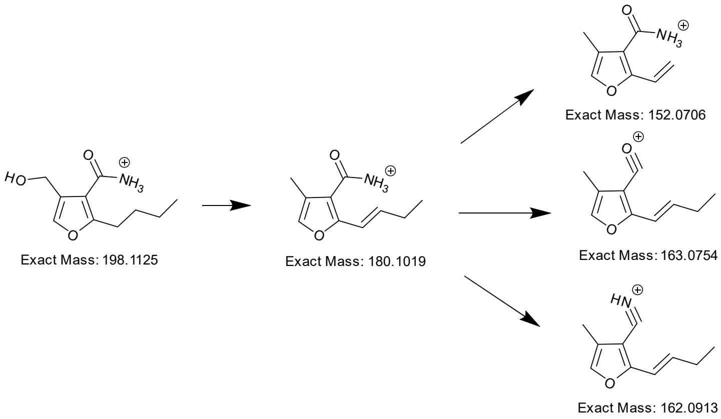

Figure S17. HR ESIMS/MS and porposed fragmentation pathway of AHFA **1.**

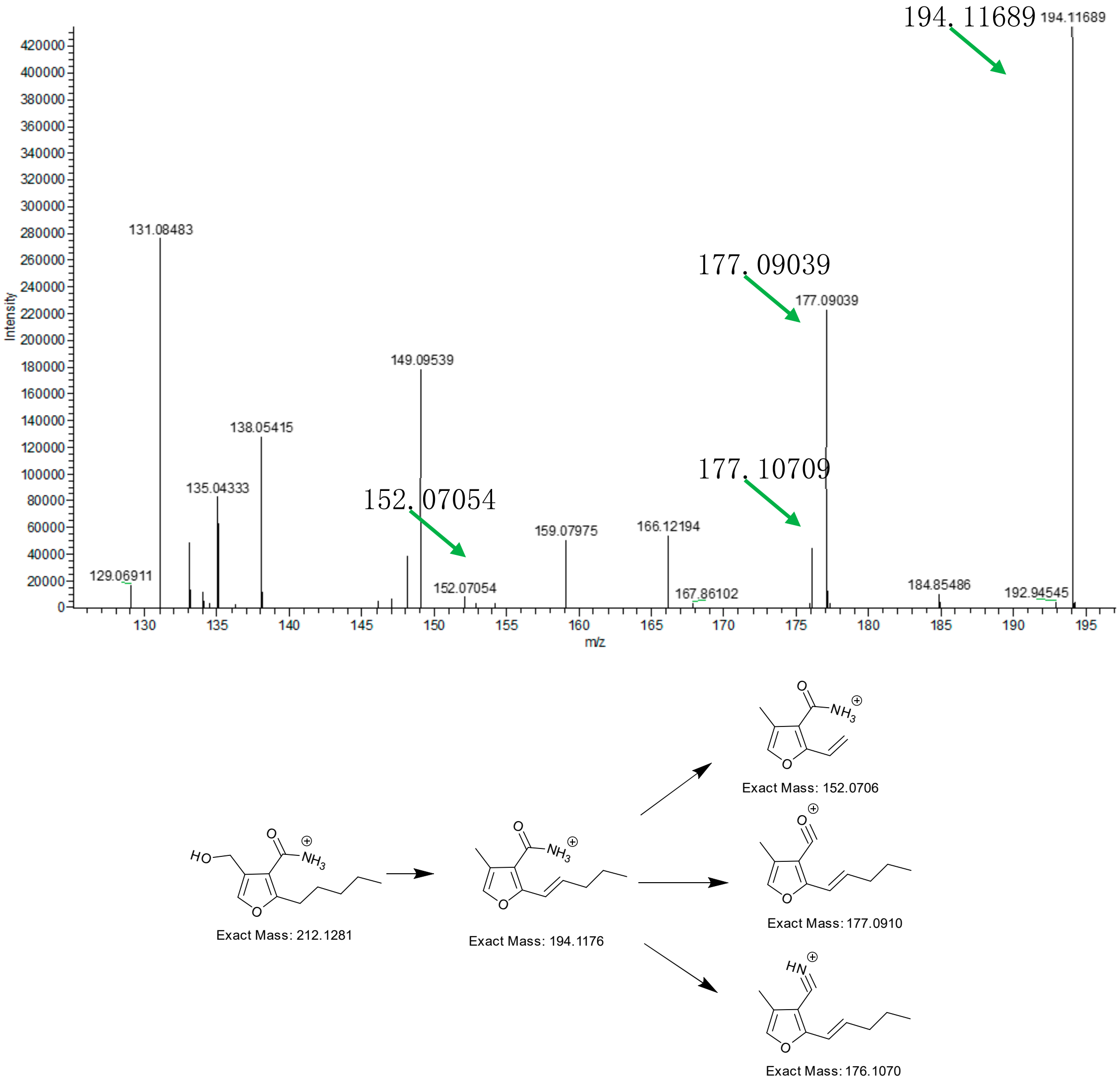

Figure S18. HR ESIMS/MS and porposed fragmentation pathway of AHFA **9.**

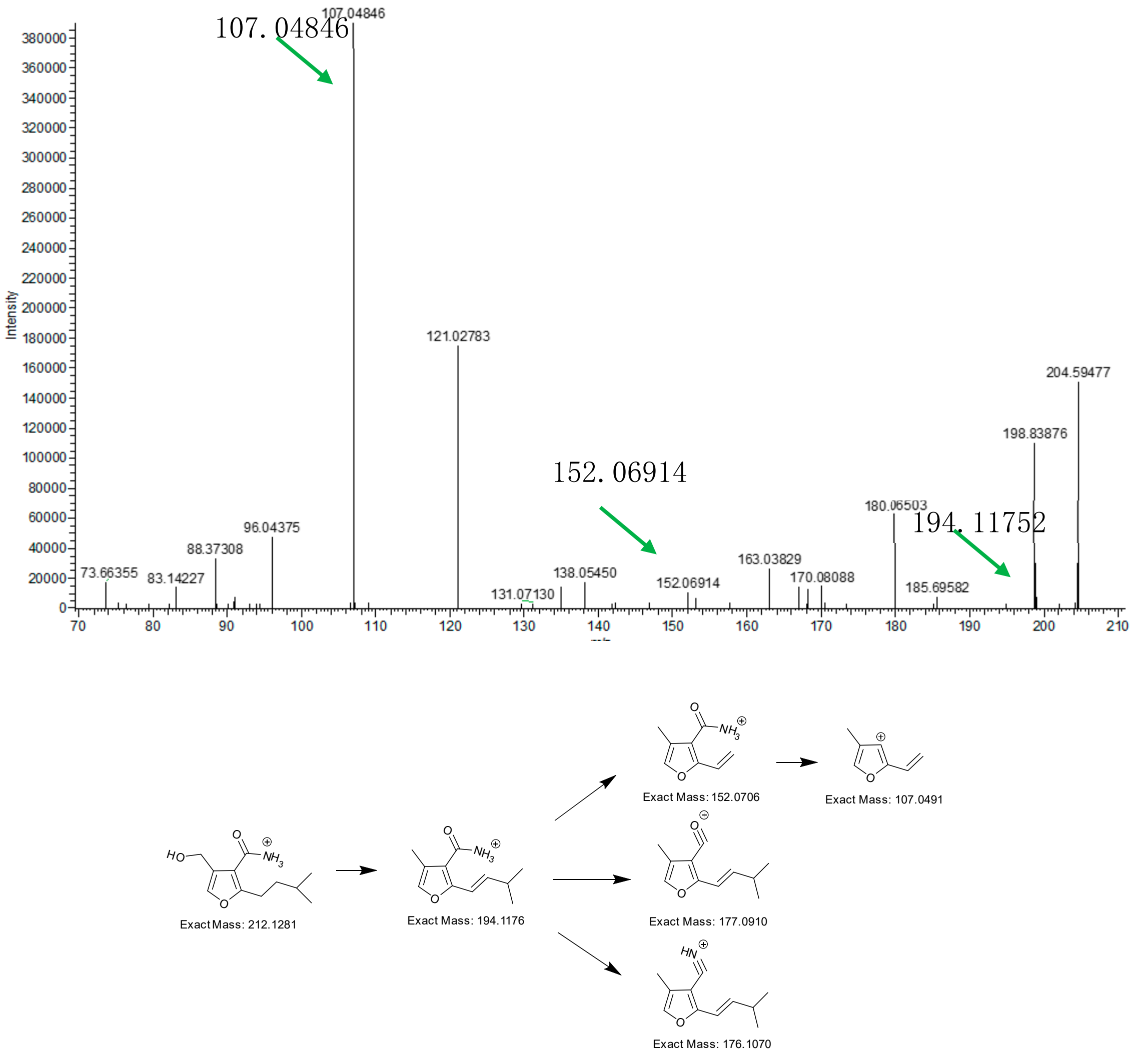

Figure S19. HR ESIMS/MS and porposed fragmentation pathway of AHFA **10.**

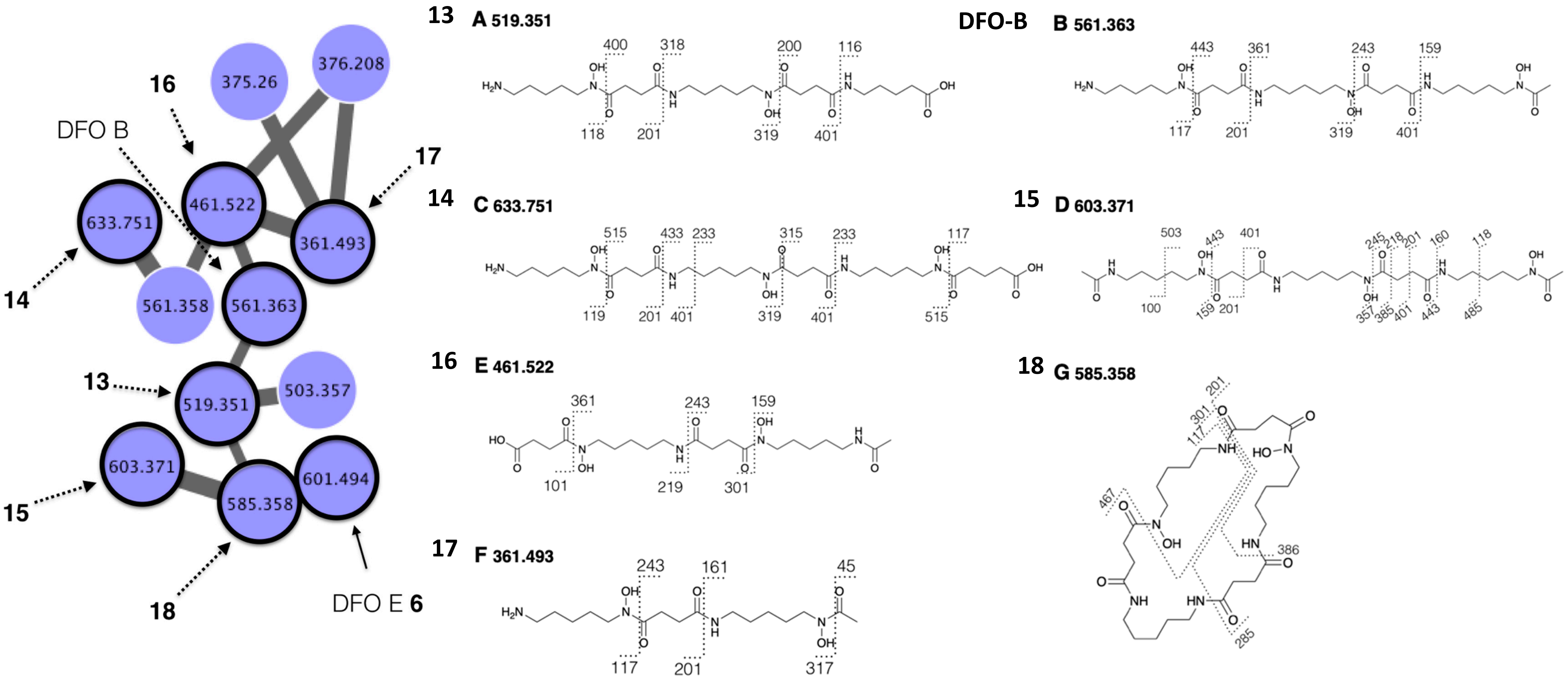

Figure S20. Siderophore molecular network, structures, and fragments. Given are the denotations, masses of protonated molecules [M+H]+, and the *m*/*z* of detected fragments. Dashed lines indicate fragmentation sites.

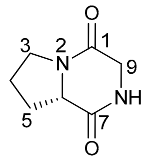

Cyclo-(Pro-Gly) **2**

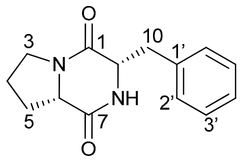

Cyclo-(Pro-Phe) **3**

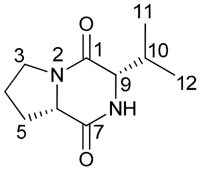

Cyclo-(Pro-Val) **4**

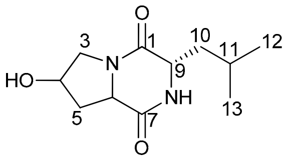

Cyclo-(Leu-Hyp) **5**

Table S2. ^{1}H and ^{13}C-NMR of **2-5** in DMSO-d_6 (^{1}H-NMR at 400 MHz and ^{13}C-NMR at 100 MHz).

No.	Cyclo-[Pro-Gly] 2		Cyclo-[Pro-Phe] 3		Cyclo-(Pro-Val] 4		Cyclo-[Leu-Hyp] 5	
	**^{13}C**	**^{1}H, mult. (J, Hz)**	**^{13}C**	**^{1}H, mult. (J, Hz)**	**^{13}C**	**^{1}H, mult. (J, Hz)**	**^{13}C**	**^{1}H, mult. (J, Hz)**
1	165, C	-	167.3, C	-		-	167.7, C	-
3	44.9, CH_2	3.49-3.62, m	44, CH_2	3.6, m<br>3.4, m	45.0, CH_2	3.35, m<br>3.36, m	53.9, CH_2	3.67, dd (13.5, 4.5)<br>3.40, d (13.5)
4	21.7, CH_2	a 2.0<br>b 1.9	23.8, CH_2	1.90-2.14, m	22.0, CH_2	1.95-2.04, m	67.8, CH	4.49, m
5	27.9, CH_2	2.29-2.35, m<br>1.95-2.03, m	27.6, CH_2	1.2, m<br>2.1, m	28.4, CH_2	2.10, m<br>1.88,m	36.6, CH_2	2.29, dd (13, 6.5)<br>2.11, ddd (13, 6.5)
6	58.4, CH	4.23, tr	57.9, CH	4.1, dd (2.7, 10.5)	58.4, CH	4.12, t,6.7	57.4, CH	4.55, dd (8.5, 4.0)
7	170.6, C	-	171.3, C	-			171.7, C	-
9	45.5, CH_2	4.1, dd (16.8)<br>3.7, d (16.9)	55.6 CH	4.5, dd(7.6)	60.2, CH	3.93, m	52.8, CH	4.20, m
10	-	-	36.1, CH_2	3.2, dd	40.4, CH	2.35, dq	38.0, CH_2	1.93, m<br>1.54, m
11	-	-	-	-	17.2, CH_3	0.94, d (6.9)	24.5, CH	1.89, m
12	-	-	-	-	18.5, CH_3	1.13, d (7.3)	21.0, CH_3	0.99, d (6.5)
13							21.6, CH_3	0.97, d (6.5)
2′	-	-	129, CH_2	7.24, m		-		
3′	-	-	127, CH_2	7.31, m		-		
4′	-	-	126, CH	7.20, m		-		

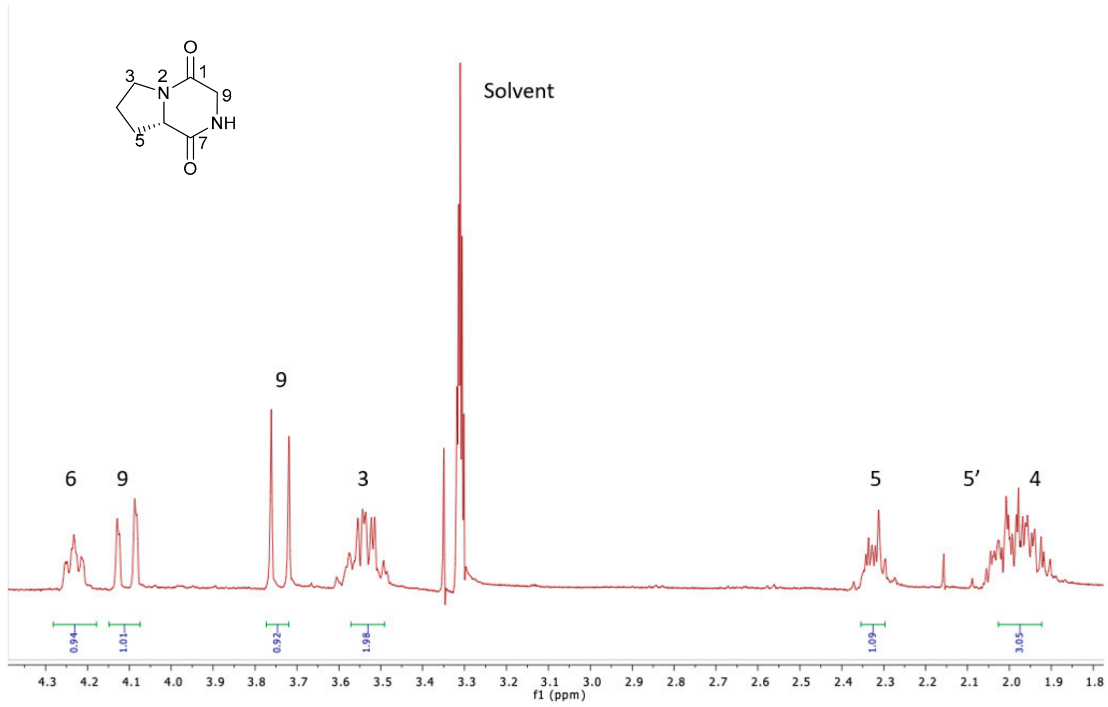

Figure S21. ^{1}H-NMR of **2** in CD_3OD (400MHz, 298K).

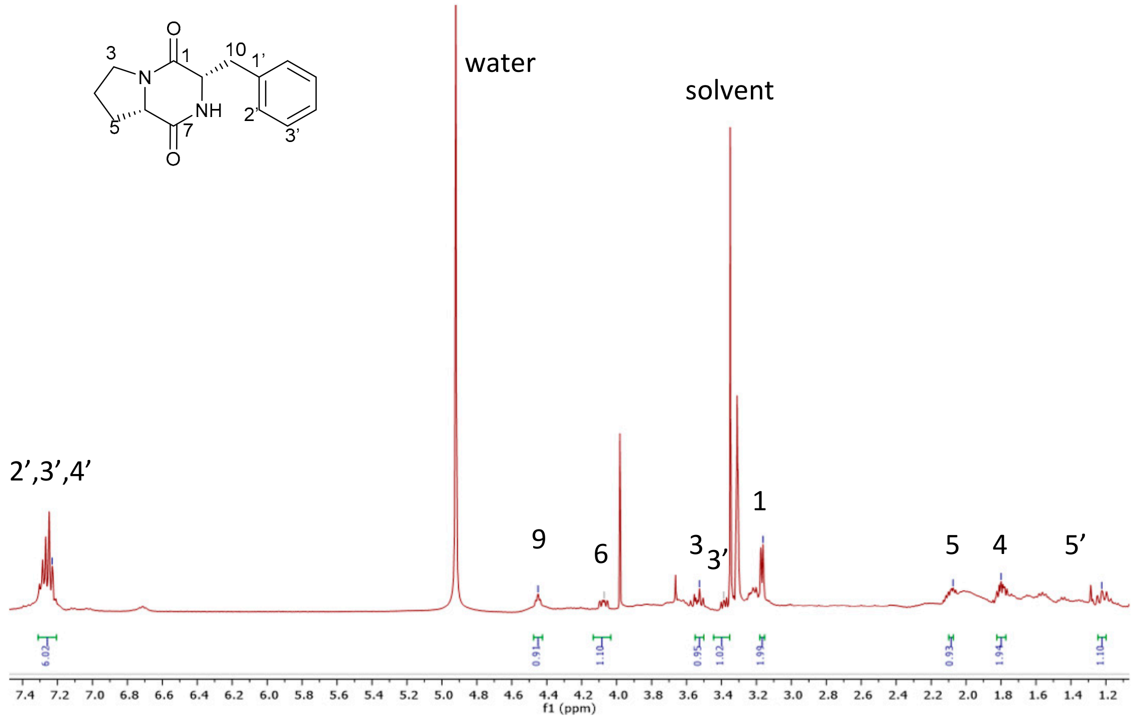

Figure S22. ^{1}H-NMR of cyclo-(L-Pro-Gly) **3** in CD_3OD (400MHz, 298K).

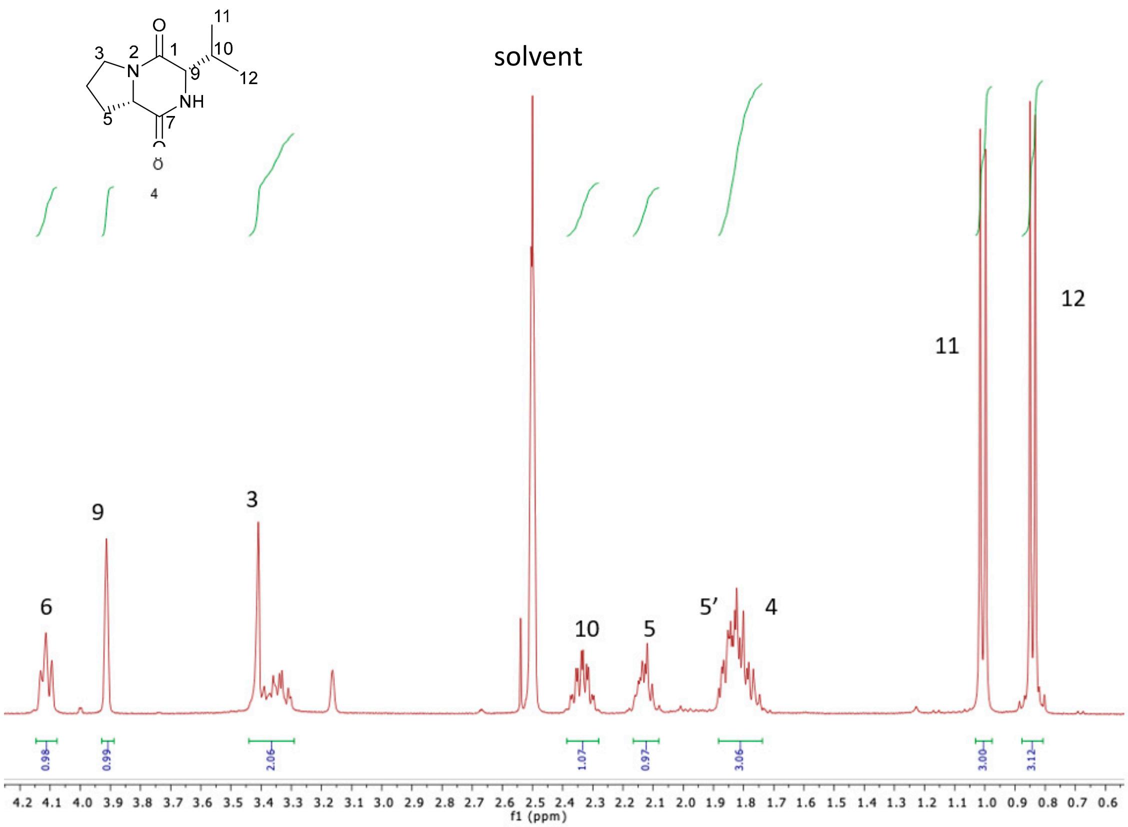

Figure S23. ^{1}H-NMR of cyclo-(L-Pro-L-Val) **4** in CD_3OD (400MHz, 298K).

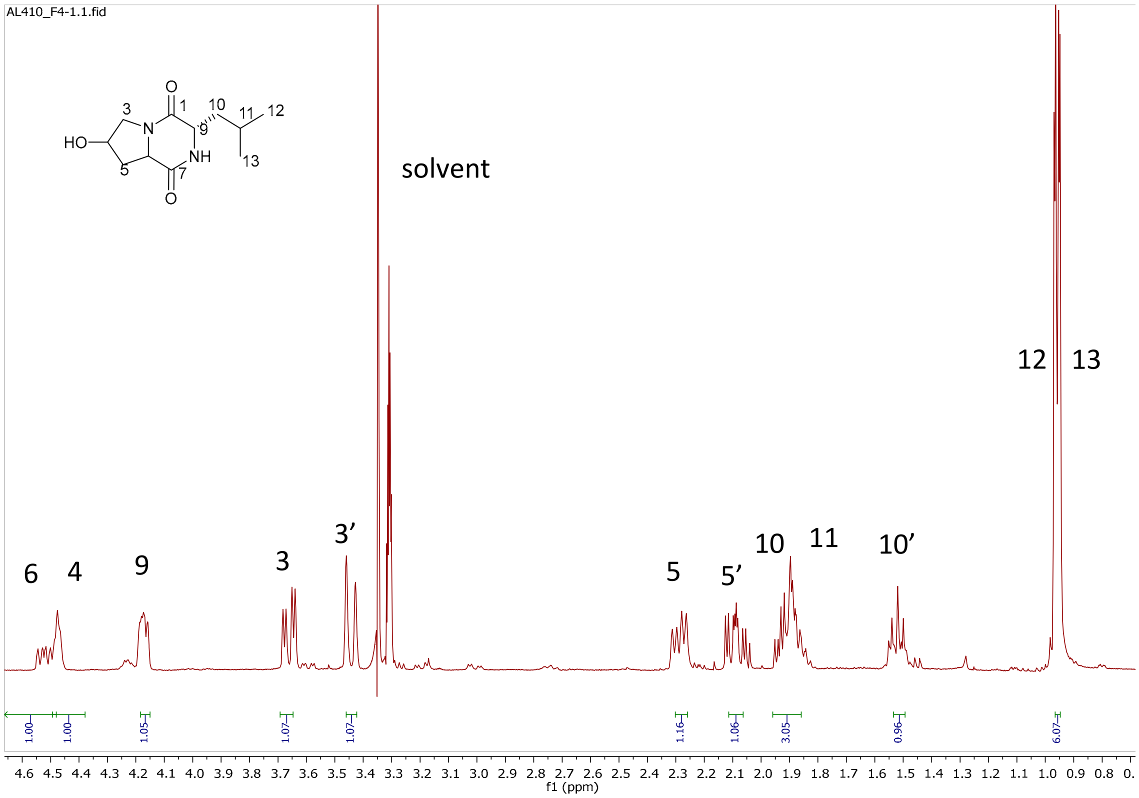

Figure S24. ^{1}H-NMR of cyclo-(L-Leu-Hyp) **5** in CD_3OD (400MHz, 298K).

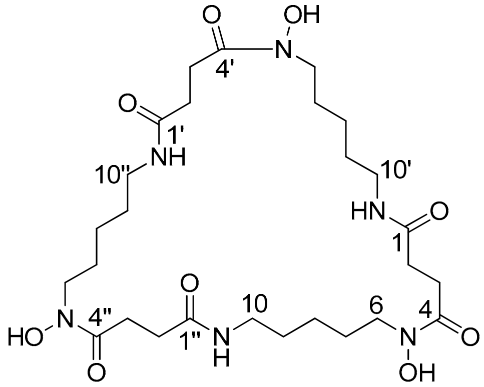

Table S3. ^{1}H and ^{13}C-NMR of deferoxamine E **6** in DMSO-d_6 (^{1}H-NMR at 400 MHz and ^{13}C-NMR at 100 MHz).

Position	^{13}C	^{1}H, mult.
1, 1′, 1′′	172.1, C	-
2, 2′, 2′′	29.9, CH_2	2.27, t
3, 3′, 3′′	27.5, CH_2	2.50, t
4, 4′, 4′′	172.4, C	-
6, 6′, 6′′	46.8, CH_2	3.46, t
7, 7′, 7′′	25.8, CH_2	1.65, m
8, 8′, 8′′	23.1, CH_2	1.30, m
9, 9′, 9′′	28.6, CH_2	1.55, m
10, 10′, 10′′	39.0, CH_2	3.01, q
N-OH	-	9.60, 3H, br
NH	-	7.72, 3H, br

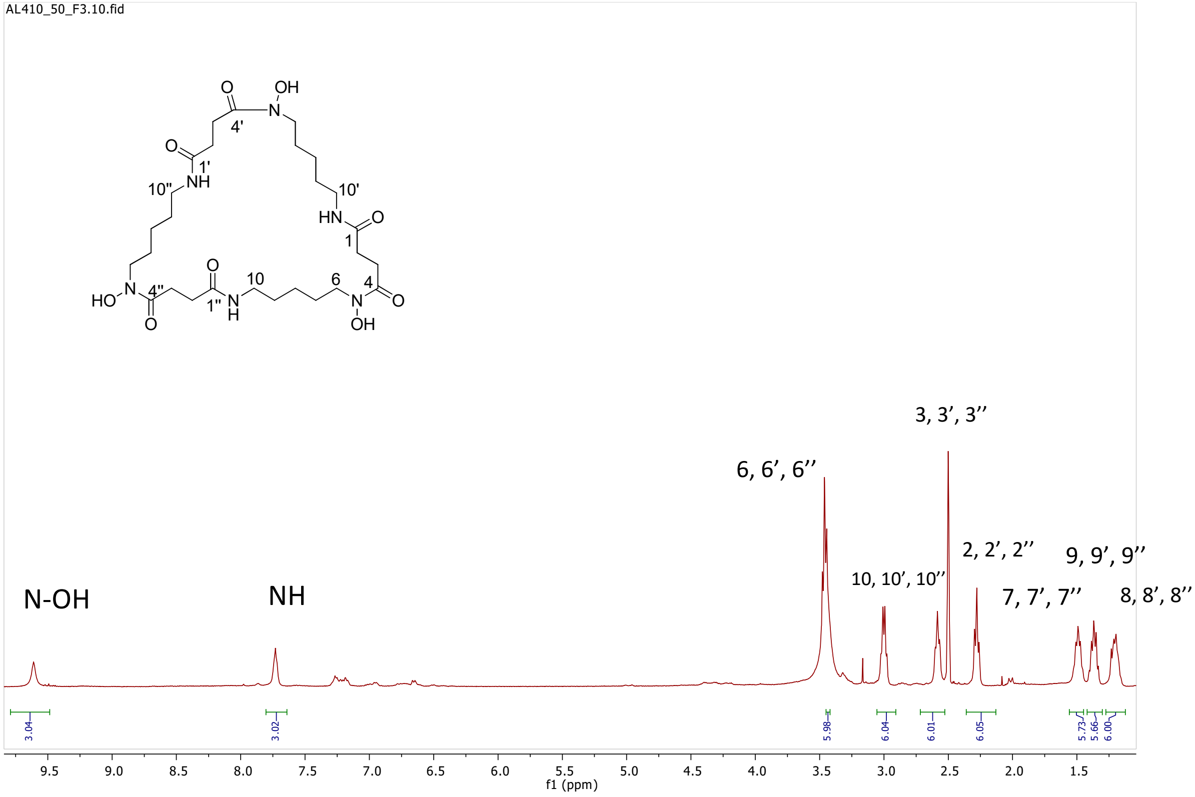

Figure S25. ^{1}H-NMR of deferoxamine E **6** in CD_3OD (400MHz, 298K).

Table S4. Advanced Marfey’s analysis of **2-5.**

**Amino Acid Derivative**	**Retention Time (min)**				
	**L-configuration AA**	**2**	**3**	**4**	**5**
Valine	11.74	-	-	11.70	-
Proline	9.34	9.34	9.32	9.30	-
Phenylalanine	12.50	-	12.48	-	-
Leucine	12.57	-	-	-	12.61

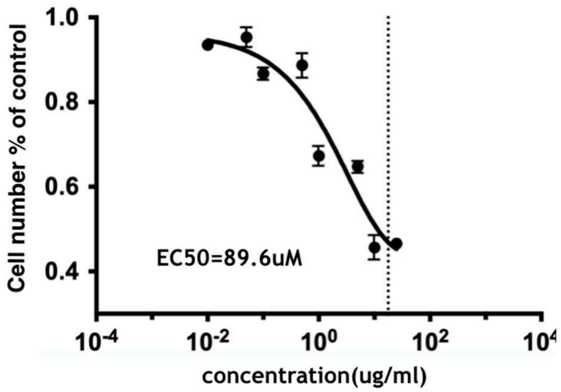

Figure S26. EC_{50} dose response curve of AHFA **1** against A2058 cancer cell line CRL-11147TM using GraphPad Prism 7.

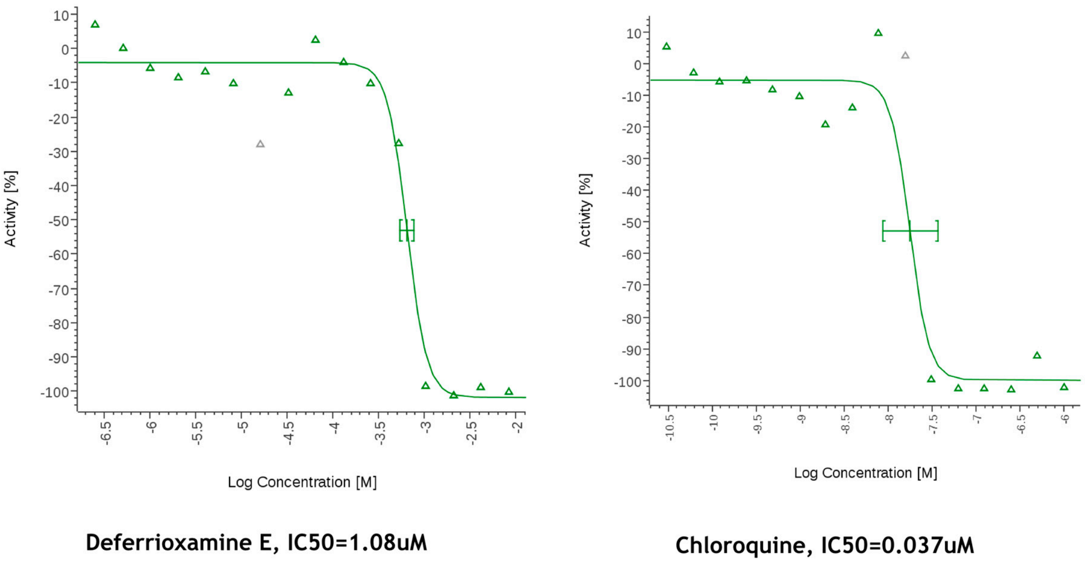

Figure S27. IC_{50} curves of DFO-E and Chloroquine against *P. falciparum* 3D7.

References:

1. Corre, C.; Song, L.; O'Rourke, S.; Chater, K.F.; Challis, G.L. 2-Alkyl-4-hydroxymethylfuran-3-carboxylic acids, antibiotic production inducers discovered by *Streptomyces coelicolor* genome mining. *Proc. Natl. Acad. Sci. U. S. A.* **2008**, *105*, 17510–17515.
2. Corre, C.; Haynes, S.W.; Malet, N.; Song, L.; Challis, G.L. A butenolide intermediate in methylenomycin furan biosynthesis is implied by incorporation of stereospecifically ^{13}C-labelled glycerols. *Chem. Commun.* **2010**, *46*, 4079–4081.
